# Bioprospecting of fungal endophytes from *Oroxylum indicum* (L.) Kurz with antioxidant and cytotoxic activity

**DOI:** 10.1371/journal.pone.0264673

**Published:** 2022-03-17

**Authors:** Nilesh Rai, Priyanka Kumari Keshri, Priyamvada Gupta, Ashish Verma, Swapnil C. Kamble, Santosh Kumar Singh, Vibhav Gautam

**Affiliations:** 1 Centre of Experimental Medicine and Surgery, Institute of Medical Sciences, Banaras Hindu University, Varanasi, India; 2 Department of Technology, Savitribai Phule Pune University, Ganeshkhind, Pune, India; Bangabandhu Sheikh Mujibur Rahman Agricultural University, BANGLADESH

## Abstract

*Oroxylum indicum* (L.) Kurz, a medicinal plant, shows numerous pharmacological properties which may be attributed to the bioactive compounds produced by *O*. *indicum* or due to associated endophytes. In the present study, leaf of *O*. *indicum* was evaluated for the presence of associated fungal endophytes, and antioxidant and cytotoxic activities of bioactive compounds produced from them. Using culture-dependent approach, eight fungal endophytes belonging to five different genera were identified. Two endophytes *Daldinia eschscholtzii* and *Ectophoma multirostrata* have been reported for the first time from the leaf of *O*. *indicum* plant. High-performance thin-layer chromatography (HPTLC) of ethyl acetate (EA) extract of isolated fungal endophytes showed a distinct fingerprinting profile in EA extract of *Colletotrichum gloeosporioides*. Among identified endophytes, EA extract of *C*. *gloeosporioides* showed significant antioxidant activity against DPPH free radical, superoxide anion radical, nitric oxide radical and hydroxyl radical with EC_50_ values of 22.24±1.302 μg/mL, 67.46±0.576 μg/mL, 80.10±0.706 μg/mL and 61.55±1.360 μg/mL, respectively. EA extract of *C*. *gloeosporioides* exhibited potential cytotoxicity against HCT116, HeLa and HepG2 cancer cell lines with IC_50_ values of 76.59 μg/mL, 176.20 μg/mL and 1750.70 μg/mL, respectively. A comparative HPTLC fingerprinting and the antioxidant activity of *C*. *gloeosporioides* associated with two different hosts (leaf of *O*. *indicum* and dead twigs of other plant) showed that *C*. *gloeosporioides* produces bioactive compounds in a host-dependent manner.

## Introduction

Endophytes are generally bacterial or fungal species, colonize into inter and intracellular tissues of the host plant and exhibit mutualistic interaction [1, 2]. Most of the fungal endophyte belongs to Ascomycota and has been reported to be associated with different tissues of the plant in natural ecosystems. With co-evolution, fungal endophytes and their respective host plant have adapted to the environmental extremities by building host-endophyte relationship [3]. During host-endophyte relationship, fungal endophyte helps to increase the fitness of the host plant against abiotic and biotic stress through horizontal gene transfer [4]. Among endophytic microbial species, fungal endophyte shows asymptomatic relationship with plant and have ability to mimic the bioactive compounds similar to their host plant. Bioactive compounds derived from fungal endophytes are of great significance and used in drug discovery and development, food industry and agriculture [5–7]. In order to preserve the biodiversity and ethnopharmacological significance of medicinal plants, fungal endophytes are explored for an alternative source for the extraction of pharmacologically significant bioactive compounds. Increasing body of evidences have shown that bioactive compounds derived from predominant fungal genera such as *Alternaria*, *Aspergillus*, *Cladosporium*, *Colletotrichum*, *Curvularia*, *Diaporthe*, *Penicillium* and *Fusarium* exhibited significant activity against oxidative stress related molecules and cancer cells [8, 9]. Thus, isolation of fungal endophytes from medicinal plants and exploring the biological properties of fungal endophyte derived bioactive compounds is an advancing approach.

*Oroxylum indicum* (L.) Kurz (also known as Mid-night Horror, Sonapatha, Shivnak, Shyonaka) is a medium-sized deciduous tree that belongs to Bignoniaceae family. It is distributed in tropical and sub-tropical regions, native to the Indian subcontinent and found in the foothills of Himalaya with a part extending to Southern China, Malaysia ecozone and Bhutan [10]. *O*. *indicum* plant is widely used in the management of various disease either alone or in combination with other drugs [11]. In prediabetic mice, the seed extract of *O*. *indicum* and acarbose have been used in combination to diminish the risk of diabetes with low toxic effects. It was also observed that combined drug of seed extract of *O*. *indicum* and acarbose significantly lowers the level of glucose than acarbose only and *O*. *indicum* only treated group [12]. The alcoholic, petroleum ether and n-butanol extract of root bark of *O*. *indicum* is used in fever, vomiting, dysentery, inflammation, asthma, intestinal worms, bronchitis and diarrhea [13, 14]. Oral consumption of seeds is also helpful in the treatment of hypertension, fever, respiratory problems and throat infection [15, 16]. Study also suggests that treatment of HeLa cells with methanolic extract of leaf showed significant antiinflammatory activity by inducing cell cycle arrest at G1/S phase and thereby causing apoptosis [17]. Few active compounds such as oroxylin A, dihydro oroxylin A, baicalein, chrysin and 7-O-methylchrysin were extracted from *O*. *indicum* with gastroprotective activity [18]. Various studies showed solvent-solvent extraction approach for isolation and characterization of bioactive metabolites from extract of stem, bark, fruit, seed, root and leaves of *O*. *indicum* plant with several biological activities, but little work has been carried out to explore fungal endophytes isolated from *O*. *indicum* and bioactive compounds with antioxidant and cytotoxic activity derived from them.

In this study, screening and isolation of fungal endophytes associated with the leaf of *O*. *indicum* (L.) Kurz have been carried out using morphological and molecular approach. High-Performance Thin Layer Chromatography (HPTLC) fingerprinting revealed that *Colletotrichum gloeosporioides* produces a unique bioactive component with distinct profile. Our results clearly show that ethyl acetate (EA) extract of *C*. *gloeosporioides* showed maximum antioxidant activity against synthetically generated free radicals, reactive oxygen radicals and nitrogen radicals. EA extracts of *C*. *gloeosporioides* possessed significant antioxidant activity due to the presence of increased content of phenolic and flavonoid compound as compared to the other fungal extracts. We have also assessed the cytotoxic activity of EA extract of all the fungal endophytes against three cancer cell lines HCT116, HeLa and HepG2 by 3-(4,5-dimethylthiazol-2-yl)-2,5-diphenyl tetrazolium bromide (MTT) assay. EA extract of *Colletotrichum* sp. WF134 showed cytotoxic activity against colorectal cancer cell (HCT116) and metastatic breast cancer cell (HeLa) only. While, EA extract of *C*. *gloeosporioides* showed cytotoxic activity against HCT116, HeLa and liver cancer cell (HepG2). In order to check the host-dependent production of fungal endophyte derived bioactive compounds, we have performed a comparative study on antioxidant activity and HPTLC fingerprinting profile of EA extract of fungal endophyte *C*. *gloeosporioides* isolated from the leaf of *O*. *indicum* and dead twig of other plant. Overall, our results show the bioprospecting of bioactive compounds derived from fungal endophytes associated with the leaf of medicinal plant *O*. *indicum* (L.) Kurz.

## Material and methods

### Collection of plant material

Leaf samples of *Oroxylum indicum* (L.) Kurz plant were collected from the Botanical Garden of Department of Dravyaguna, Institute of Medical Sciences, Banaras Hindu University, Varanasi, India. Samples were collected in the sterile polybag and isolation procedure of fungal endophytes was performed within 48 hours of collection.

### Isolation and morphological identification of fungal endophytes

The leaf samples were washed under running tap water followed by deionized water and surface disinfection was done by following standard method with minor modifications [7, 19]. Briefly, the leaf samples were immersed in 70% ethanol for 1 minute, then in 0.1% sodium hypochlorite (NaOCl; Biochem, India) for 1 minute, dipped in 70% ethanol for 1 minute followed by washing with distilled water twice for 1 minute. Samples were air-dried under the sterile condition and cut into smaller pieces of 1 cm^2^ size. These leaf segments were evenly placed onto potato dextrose agar (PDA) (SRL, India) amended with 250 μg/mL streptomycin (SRL, India), used to suppress bacterial contamination. Petri dish was sealed with Parafilm^™^ and incubated at 27±2 °C for 7–8 days in biological oxygen demand (BOD) incubator. The mycelial growth was carefully monitored and the hyphal tips of each fungus that grew out were sub-cultured onto a fresh PDA plate for 9–10 days at 27±2 °C to isolate a pure colony of individual fungal strains.

For morphological characterization of the fungal isolates, semi-permanent slides were prepared by taking the mycelia of fungal strains from PDA plates on a slide and stained with Lacto-phenol cotton blue [20, 21]. Observations were made under bright field microscope (Olympus, CX43, Japan).

### Molecular identification of fungal endophytes

The molecular examination of pure colonies was performed according to the standard method as described previously [22]. Briefly, mycelium grown onto the sub-cultured PDA plate was transferred into a tube containing 25 mL potato dextrose broth medium supplemented with 250 μg/mL streptomycin (SRL, India). Cultures of isolated fungal strains were inoculated at 27±2 °C for 5–7 days in shaker incubator (Metrex, India) at 120 rpm. After growth of mycelium the genomic DNA was isolated using Nucleo-pore gDNA Fungal mini kit (NP-7006D) according to the manufacturer protocol. The concentration of isolated gDNA of each strain was quantified using NanoDrop ONE (AZY1811395, Thermo Scientific, USA).

The extracted gDNA was used for polymerase chain reaction (PCR) amplification. The primers used in PCR were of conserved internal transcribed spacer region, ITS1 (5’-TCCGTAGGTGAACCTGCGG-3’), ITS4 (5’-TCCTCCGCTTATTGATATGC-3’) and ITS5 (5’-GGAAGTAAAAGTCGTAACAAGG-3’) in different combination for each strain. The amplification was performed for 20 μL solution, each containing 2 μL of extracted genomic DNA as template, 10 μM forward and reverse primer of 1.5 μL each, 2 μL of 10X buffer, 0.5 μL of *Taq* polymerase enzyme (BR Biochem, India), 0.75 μL of 10 mM dNTP mix (BR Biochem, India) and volume was makeup with milli-Q water. For thermal cycle PCR, following parameters were used: 94 °C for 5 minutes followed by elongation for 35 cycles of 30 s at 94 °C, 40 s at 60 °C and 1 minute at 74 °C and a final extension at 74 °C for 10 minutes.

Amplification was checked by agarose gel electrophoresis using 1% agarose gel in 1X TAE buffer (**S2 Fig in** S1 File). PCR product was purified using the Nucleopore Quick PCR purification kit (NP-36105) according to the manufacturer protocol. Sequencing for the purified PCR product was performed using ITS1, ITS4 and ITS5 primers in different combinations.

### Sequence alignment and phylogenetic analysis

To identify the fungal endophytes, consensus DNA sequences were used, while the raw sequences (obtained after sequencing) of internal transcribed spacer region of fungal isolates were used for constructing a phylogenetic tree [23]. The overlapping common sequence was identified through the sequence alignment between FP-ITS and RP-ITS sequences for each isolate. The common sequences constructed for all isolates were used as a query to NCBI-BLAST (http://www.ncbi.nlm.nih.gov/BLAST/). The fungal endophytes and their consensus DNA sequences were identified by comparing the query sequence in NCBI-BLAST with the previously submitted sequences in GenBank and designated with accession number. In the case of common overlapping sequence not available for an isolate, both the FP-ITS and RP-ITS sequences were used separately. Multiple consensus sequences for the fungal isolate OI-L2 and OI-L7 has been found. These consensus DNA sequences were used in carrying out phylogenetic study.

Multiple Sequence Alignment (MSA) of the sequences was performed using the Multiple Sequence Comparison by Log-Expectation (MUSCLE) program. Percentage identity of aligned sequences was studied using Kolmogorov-Smirnov statistical test in GeneDoc (version 2.7). The phylogenetic analysis was performed through the construction of phylogenetic tree using MEGA (v10.1.8) phylogenetic analysis tool by the maximum likelihood Bootstrap (MLBS) method.

### Fermentation and extraction of crude fungal extract

The sub-cultured fungal strains were fermented in Erlenmeyer flask each containing 200 mL potato-dextrose broth supplemented with 250 μg/mL of streptomycin. Further, flasks were kept in an orbital shaker incubator at 27±2 °C for 20–25 days at 120 rpm. After fermentation, mycelium was filtered through two-layer of cheesecloth and was dried overnight at 50 °C. Dried mycelium was macerated using liquid nitrogen and fungal residue (metabolite) was extracted with EA (5X volume). The organic phase was separated and evaporated to dryness using rotary evaporator at 30 °C [24]. The obtained crude fungal extract was stored at 4 °C for further experiments.

### Estimation of total phenolic and total flavonoid content

Total Phenolic contents (TPC) present in EA extract of fungal isolates were determined by Folin-Ciocalteu (FC) method with minor modifications [25]. Briefly, 1 mL aliquot of EA extract was added to 100 μL of 0.5 N FC reagent (diluted with water in 1:1 v/v) and allowed to stand for 15 minutes. Further, 2.5 mL of sodium carbonate was added and incubated for 30 minutes at room temperature. Post-incubation absorbance was measured at 765 nm using a UV spectrophotometer against methanol as blank. Different concentration (5–200 μg/mL) of Gallic acid was used to plot calibration curve. Total phenolic contents were expressed as μg of gallic acid equivalents (GAE) per mg of EA extract.

The estimation of total flavonoid contents (TFC) was performed through aluminium chloride (AlCl_3_.H_2_O) method [26]. Briefly, 1 mL of EA extract was mixed with 3 mL of methanol, 200 μL of 10% AlCl_3_, 200 μL of 9.8% potassium acetate. Further, the solution was diluted with 5.6 mL of distilled water. Post incubation of 30 minutes, absorbance was measured at 420 nm using UV spectrophotometer against methanol as blank. Different concentration (5–200 μg/mL) of Quercetin was used to plot calibration curve. Total flavonoid contents were expressed as μg of quercetin equivalents per mg of EA extract.

### High-Performance Thin Layer Chromatography (HPTLC) fingerprinting

HPTLC of the EA extract of fungal endophytes was performed using CAMAG HPTLC instrument (ANCHROM, Switzerland), comprises of a Linomat-4 autosampler, CAMAG TLC scanner-4 and visualizer with minor modification [27]. HPTLC finger printing was performed on 20 × 10 cm pre-coated silica gel 60 F_254_ TLC plate (Merck, Germany). EA extracts (100 μg) of each fungal endophyte were applied by Linomat-4 autosampler equipped with Hamilton syringe (100 μl) on the TLC plate with band length 8 mm. After the application of EA extract, TLC plate was placed in HPTLC Twin trough chamber with mobile phase of toluene: chloroform: ethyl alcohol (4: 4: 1, v/v/v). The resulting plate was allowed to dry at room temperature for 5 minutes. Image of plate was captured by visualizer under short UV range 254 nm and long UV range 366 nm. TLC plate was scanned by TLC scanner-4 at UV λ_max_ 254 nm (D2 light source) and 366 nm (Hg lamp, fluorescence mode), and area percentage and value of retention factor (R_f_) for the separated components was analyzed by winCATS software (version 1.4.10.0001). Post-chromatographic derivatization was performed with anisaldehyde sulphuric acid reagent (ASR).

### Antioxidant activity of EA extract of identified endophytic fungi associated with the leaf of *Oroxylum indicum* (L.) Kurz

#### Free radical scavenging assay

2,2-diphenyl-1-Picrylhydrazyl (DPPH)-radical scavenging activity of crude fungal extract was performed as described previously with minor modifications [28]. Briefly, 2 mL of EA extract with different concentration (1–200 μg/mL) was added to 2 mL of 50 μg/mL of DPPH (SRL, India) solution, and incubated in dark for 30 minutes followed by brief vortexing. Absorbance was measured at 517 nm using UV-spectrophotometer (SHIMADZU, UV-1800, Japan). Methanol (Merck, India) was used as a blank and 50 μg/mL of DPPH solution was used as a control. L-ascorbic acid (SRL, India) at different concentrations (5–100 μg/mL) was used as a positive control. The percentage of free radical scavenging potential was calculated using the following formula: [1-(Abs_(517nm)_ of the sample / Abs_(517nm)_ of the control)] x 100.

#### Superoxide anion scavenging assay

The superoxide anion scavenging assay of crude fungal extract was performed as described previously with minor modifications [29]. Briefly, different concentration of EA extract (1–200 μg/mL) was added to 1 mL of 150 μM Nitroblue Tetrazolium (NBT) solution (SRL, India) in 1X phosphate buffer saline (PBS) pH 7.4 and 1 mL of 468 μM NADH solution in 1X PBS. Further, 100 μL of 60 mM Phenazine methosulphate solution (SRL, India) dissolved in 1X PBS pH 7.4 was added to initiate the reaction and the solution was incubated for 5 minutes at 25 °C. Absorbance was measured at 560 nm using UV spectrophotometer, a mixture of NBT, NADH and PMS was used as a control and methanol as a blank. L-ascorbic acid at different concentrations (5–100 μg/mL) was used as a positive control. The percentage of superoxide anion scavenging potential was calculated using the following formula: [1-(Abs_(560nm)_ of the sample / Abs_(560nm)_ of the control)] x 100.

#### Nitric oxide scavenging assay

EA extract with different concentrations (1–200 μg/mL) was added in 150 μL of 10 mM Sodium nitroprusside (SRL, India) dissolved in PBS and the mixture was incubated for 150 minutes. Post-incubation, 200 μL Griess reagent (SRL, India) was added to the 200 μL of reaction mixture and allowed to react for 30 minutes. Absorbance was measured at 546 nm against methanol blank using UV spectrophotometer. The percentage of nitric oxide scavenging potential was calculated using the following formula: [1-(Abs_(546nm)_ of the sample / Abs_(546nm)_ of the control)] x 100 [24]. L-ascorbic acid at different concentrations (5–100 μg/mL) was used as a positive control.

#### Hydroxyl radical scavenging assay

A reaction mixture of 3.75mM of 2-deoxyribose (SRL, India), 100mM of EDTA, 100 μM of FeCl_3_ (Merck) and 1mM of H_2_O_2_ (Qualigens Fine Chemicals, India) was prepared and added to EA extract with different concentrations (1–200 μg/mL). The reaction mixture was incubated for 1 hour at 37 °C. Post-incubation, 1 mL of 2% Trichloro-acetic acid (TCA) (SRL, India) and 1 mL of 1% Thiobarbituric acid (TBA) (SD Fine Chemicals, India) were added and further incubated in a water bath at 90 °C for 20 minutes. After incubation, the solution was cooled and absorbance was measured at 417 nm using UV spectrophotometer. Methanol was used as a blank and reaction mixture (2-deoxyribose, EDTA, FeCl_3_, and H_2_O_2_) was used as a control. The percentage of hydroxyl radical scavenging potential was calculated using the following formula: [1-(Abs_(417nm)_ of the sample / Abs_(417nm)_ of the control)] x 100 [24]. L-ascorbic acid at different concentrations (5–100 μg/mL) was used as a positive control.

### Cell culture maintenance and cytotoxicity assay

Authenticated cell lines HEK 293T (non-cancerous human embryonic kidney cells), HCT116 (human colorectal carcinoma cell line), HepG2 (human liver carcinoma cells) and HeLa (metastatic breast cancer cell line) were procured from National Centre for Cell Science, Pune, India, and maintained as described previously [30]. Cytotoxic activity of EA extract of identified fungal isolates was performed using 3-(4,5-dimethylthiazol-2-yl)-2,5-diphenyl tetrazolium bromide (MTT) assay [31]. The cells were grown in Dulbecco’s modified Eagle’s medium (DMEM) for HEK 293T, HCT116 and HepG2, or Minimum Essential Medium (MEM) for HeLa cells with 10% fetal bovine serum (FBS) in T-25 flask and were passaged using trypsin-EDTA upon 80–90% confluence. Cells were maintained in 5% CO_2_ incubator at 37 °C and observed daily for morphology and presence of any contaminant. For cell proliferation assay, 5 x 10^4^ cells/well were seeded in a 96 well plate and incubated overnight at 37 °C in 5% CO_2_ incubator. The cells were treated with extract having concentrations range of 0–2000 μg/mL or doxorubicin (D1515, Sigma-Aldrich, MA, USA), or drug vehicle control (DMSO) and left overnight in incubator. Next day, 5 mg/mL of MTT (219459291, MP Biomedicals, USA) per well was added and incubated for 2 hours at 37 °C. Formazan crystals hence formed, were solubilized by adding 100 μL DMSO and incubated for 10 minutes at room temperature in dark. Absorbance was measured at 570 nm with reference at 630 nm using Multiskan microplate spectrophotometer (ThermoFisher Scientific, USA).

### Statistical analysis

All the experiments of antioxidant and cytotoxicity were performed in triplicate (n = 3). EC_50_ value for antioxidant assays were calculated using Graph Pad Prism 8.0.2 software. EC_50_ value indicates the concentration of EA extract of fungal endophytes required to scavenge 50% of the oxidative free radicals. Data for antioxidant assays are presented as mean ± S.D in histogram. For the statistical significance of antioxidant data, means ± S.D. of all groups were compared and One-Way ANOVA (analysis of variance) was performed using Graph Pad Prism 8.0.2 software. Analysis for cytotoxicity data and IC_50_ values were calculated by using Origin software version 9.8. IC_50_ value indicates the concentration of EA extract of fungal endophytes required to kill 50% of the cancer cells.

## Results and discussion

### Eight fungal endophyte strains were isolated using culture dependent approach

**Fig 1:**
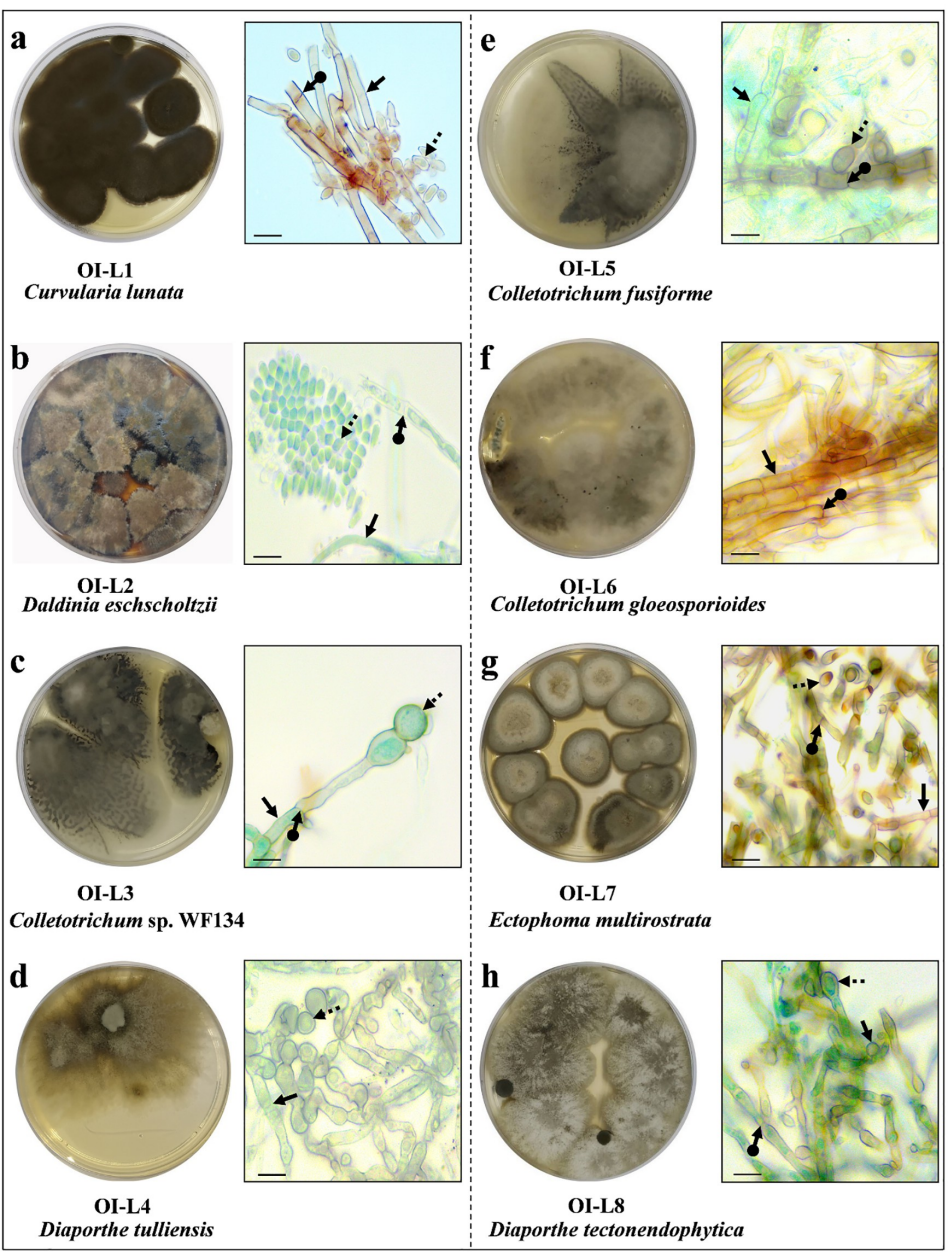
Photomicrograph showing the morphology of fungal colony on PDA plate after 10 days at 27±2 °C, corresponding light microscope image of fungal endophytes **(a)** *C*. *lunata*, **(b)** *D*. *eschscholtzii*, **(c)** *Colletotrichum* sp. WF134, **(d)** *D*. *tulliensis*, **(e)** *C*. *fusiforme*, **(f)** *C*. *gloeosporioides*, **(g)** *E*. *multirostrata* and **(h)** *D*. *tectonendophytica*. Scale bar = 100 μm.

A total of eight fungal endophytes were isolated from the leaf tissue of *O*. *indicum* using culture dependent approach which were encoded as OI-L1, OI-L2, OI-L3, OI-L4, OI-L5, OI-L6, OI-L7 and OI-L8. The coding of isolated fungal strains was done in a specific manner, for example OI-L1 encodes for 1^st^ fungal isolate from the leaf of *O*. *indicum*. The isolated strains were further identified using light microscope considering the properties such as color of the colonies, morphology of the hyphae, seta, conidia and spores (Fig 1). Strain OI-L1 was identified as of *Curvularia* sp. with olive-black colony, septate hyphae, nodulose conidiophore with presence of ellipsoidal conidia, round at the ends. OI-L2 was identified as *Daldinia* sp. due to cotton like appearance which was white at the periphery to smoky grey in the middle with olivaceous tone. Hyphae were septate, often have exudates, aseptate conidia and ascospore with germ slits. However, based on identification using molecular technique, sequence of OI-L2 was similar to *D*. *eschscholtzii*, and *Nodulisporium* sp. strain MJ37 also. As both fungal strains belong to the same family Xylariaceae of the division Ascomycota, so both were denoted as OI-L2. Three isolated strain OI-L3, OI-L5 and OI-L6 were identified as of *Colletotrichum* sp. due to the presence of grey, cottony copious cinnamon masses of conidia with smooth-walled conidiophore, hyaline and unbranched. Fungal isolates OI-L6 was identified as *C*. *gloeosporioides* due to presence of white to smoky grey mycelia, smooth walled, septate, cottony hyaline cylindrical conidia and elliptic to fusoid shape appressoria. Sometimes the appressoria of *C*. *gloeosporioides* develops as broad or with irregular lobes (**S1 Fig in** S1 File). Fungal strains OI-L4 and OI-L8 showed morphological similarity with *Diaporthe* sp. due to the presence of white mycelia in the center with flat olivaceous grey mycelium in the periphery. Conidia were non-septate, hyaline, ellipsoidal, rounded, fusiform with straight or curved ends. OI-L7 showed colony features similar to *Ectophoma* sp. with olive-green to dark brown colony. The conidia were ellipsoid, non-septate, hyaline and single celled. The mycelium was aerial and compact.

### Eight fungal endophyte species belonging to the division Ascomycota were identified

**Fig 2:**
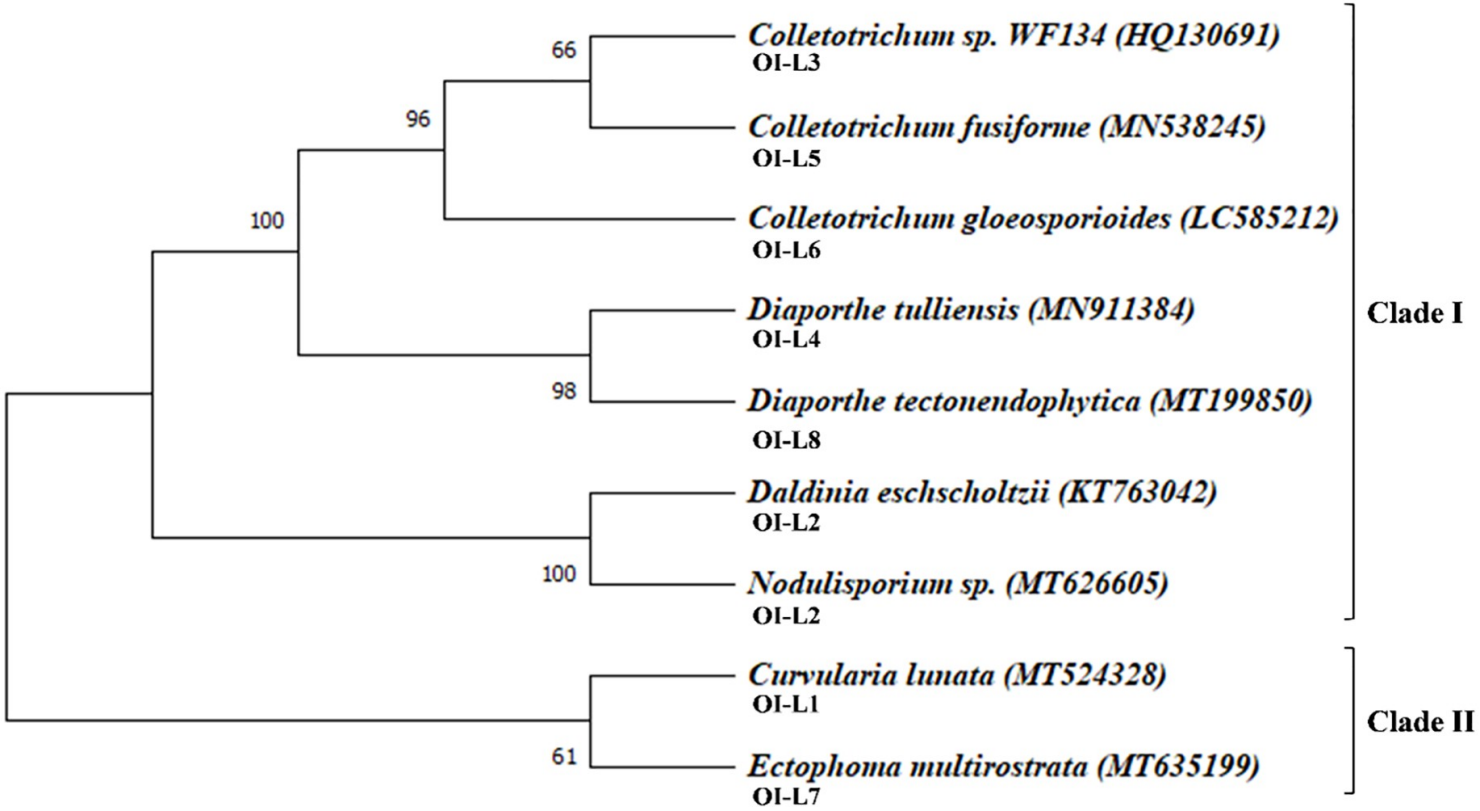
Phylogenetic tree of fungal endophytes constructed by maximum likelihood bootstrap (MLBS) method. The sequences were aligned through MUSCLE alignment program and the evolutionary history was inferred by using the Maximum Likelihood bootstrap (MLBS) method and General Time Reversible model.

Using internal transcribed spacer sequences [23] as a query in a BLAST search, we identified the strains and their complete 5.8S ribosomal RNA gene sequences (**S1 Table in** S1 File). Identified fungal strains were *Curvularia lunata*, *Daldinia eschscholtzii* or *Nodulisporium* sp. strain MJ37, *Colletotrichum* sp. WF134, *Diaporthe tulliensis*, *Colletotrichum fusiforme*, *Colletotrichum gloeosporioides*, *Ectophoma multirostrata* and *Diaporthe tectonendophytica* (named as OI-L1 to OI-L8, respectively). In order to identify fungal evolution and the taxonomic position, we reconstructed phylogenetic tree using these sequences (Fig 2). The reconstructed phylogenetic tree was clustered into two Clades; Clade-I and Clade II. The sister lineages of the clade I and II was classified into two fungal taxonomic classes under division Ascomycota: Sordariomycetes and Dothideomycetes. It was observed that the clade I consist of seven fungal strains; three of *Colletotrichum* species i.e., *C*. *fusiforme* (MN538245), *C*. *gloeosporioides* (LC585212), and an uncharacterized *Colletotrichum* sp. WF134 (HQ130691). The other sequences were of *D*. *eschscholtzii* (KT763042), *Nodulisporium* sp. strain MJ37 (MT626605) and *Diaporthe* species i.e., *D*. *tectonendophytica* (MT199850) and *D*. *tulliensis* (MN911384). Clade II branch of the phylogenetic tree consist of two species i.e., *E*. *multirostrata* (MT635199) and *C*. *lunata* (MT524328). It was inferred from the phylogenetic tree that the clade II sister members have diverged earlier from the clade I.

### EA extract of OI-L6 contains highest amount of phenolic and flavonoid content

The remarkable potential of phenolic and flavonoid compounds in counteracting the deleterious effect of free radical, label them as a powerful antioxidant. The activity is variable with respect to the amount of phenolic and flavonoid contents present in the EA extract of isolated fungal endophytes. Content for total phenolic compounds present in the EA extract of all the isolated fungal endophytes showed a 2-fold variation, ranges from 26.52 to 55.16 μg of gallic acid equivalents (GAE) per mg of EA extract (Table 1). The highest phenolic content was shown by the extract OI-L6 (*C*. *gloeosporioides*) that is, 55.16 μg GAE/mg of EA extract and also own highest antioxidant activity, while OI-L8 possessed lowest phenolic content value of 26.52 μg of GAE/mg of EA extract. The standard graph for Gallic acid represents linear relationship between concentration and absorbance with correlation coefficient, R^2^ = 0.9939 (**S3a Fig in** S1 File).

**Table 1:** Total phenolic and flavonoid content present in EA extract of identified fungal endophytes associated with the leaf of *O*. *indicum*.

Isolated strains	Species	Total Phenolic content (μg of GAE/mg of crude extract)	Total flavonoid content (μg of QE/mg of crude extract)
OI-L1	*C*. *lunata*	43.15	10.96
OI-L2	*D*. *eschscholtzii*	27.36	23.85
OI-L3	*Colletotrichum* sp. WF134	27.30	30.46
OI-L4	*D*. *tulliensis*	34.96	8.44
OI-L5	*C*. *fusiforme*	46.36	30.66
OI-L6	*C*. *gloeosporioides*	55.16	81.95
OI-L7	*E*. *multirostrata*	36.74	14.22
OI-L8	*D*. *tectonendophytica*	26.52	11.70

Total flavonoid content showed a variation of approximately 10-fold, ranges from 8.44 to 81.95 μg of QE per mg of EA extract (Table 1). The highest value for flavonoid content was exhibited by EA extract of OI-L6 (*C*. *gloeosporioides*) that is, 81.95 μg QE/mg of EA extract and also showed highest antioxidant activity, whereas OI-L8 possessed lowest flavonoid content value of 8.44 μg of QE/mg of EA extract. The linear relationship was established between the concentration of Quercetin and its absorbance, represented by standard graph for Gallic acid with R^2^ = 0.998 (**S3b Fig in** S1 File).

Extraction of metabolite using ethyl acetate is a most effective method to isolate bioactive compounds derived from fungal endophyte. Ethyl acetate is used as a solvent to selectively extract polyphenols of high molecular weight and phenolic compounds of low molecular weight [32, 33]. Results for TPC and TFC showed the presence of varying degree of phenolic and flavonoid compounds in the EA extract of isolated fungal endophytes. Out of eight isolated fungal strains, the EA extract of endophyte OI-L6 (*C*. *gloeosporioides*) contained the highest phenolic and flavonoid content. In a report, the extract of fungal isolates showed increased production of phenolics (4.577 μg RE) and flavonoids (4.111 μg RE) compounds show good agreement with the increased antioxidant activity [34]. Similarly, *Chaetomium* sp. showed 80% antioxidant activity that can be attributed to its high phenolic value i.e., 60.13±0.41 mg/GAE [32]. Similar to the previous reports, our study also reveals that EA extract of endophytes *Colletotrichum* sp. WF 134 (OI-L3) and OI-L6 (*C*. *gloeosporioides*) yields higher flavonoid content than that of phenolic content. One of the previous report suggests that EA extract of fungal endophyte *Achaetomium* sp. associated with *Euphorbia hirta* yields 44.02±1.57 μg of total phenolic content which is lower than the produced flavonoid content (54.54±1.82 μg) [35]. Furthermore, EA extract of OI-L5 (*C*. *fusiforme*) contained phenolic content of 46.36 μg GAE/mg that is somewhat closer to phenolic content exhibited by OI-L6 (*C*. *gloeosporioides*). However, the antioxidant activity was not observed to be significant for fungal extract OI-L5. This observation may be attributed due to presence of synergistic and antagonistic interactions between various phenolic compounds with antioxidant activity [36, 37]. The complete account of the quality and quantity of phenolic compounds present in the extract of fungal endophyte cannot be given by Folin–Ciocalteu method and also the presence of some interfering chemical constituents can affect the determination of total phenolic content present in an EA extract and antioxidant activity [38]. The significant antioxidant activity is validated through the higher phenolic content in the extract of fungal endophytes but the higher phenolic content cannot predict the antioxidant activity of an extract of fungal endophyte.

### HPTLC fingerprinting show the presence of more bioactive components in EA extract of OI-L6

**Fig 3:**
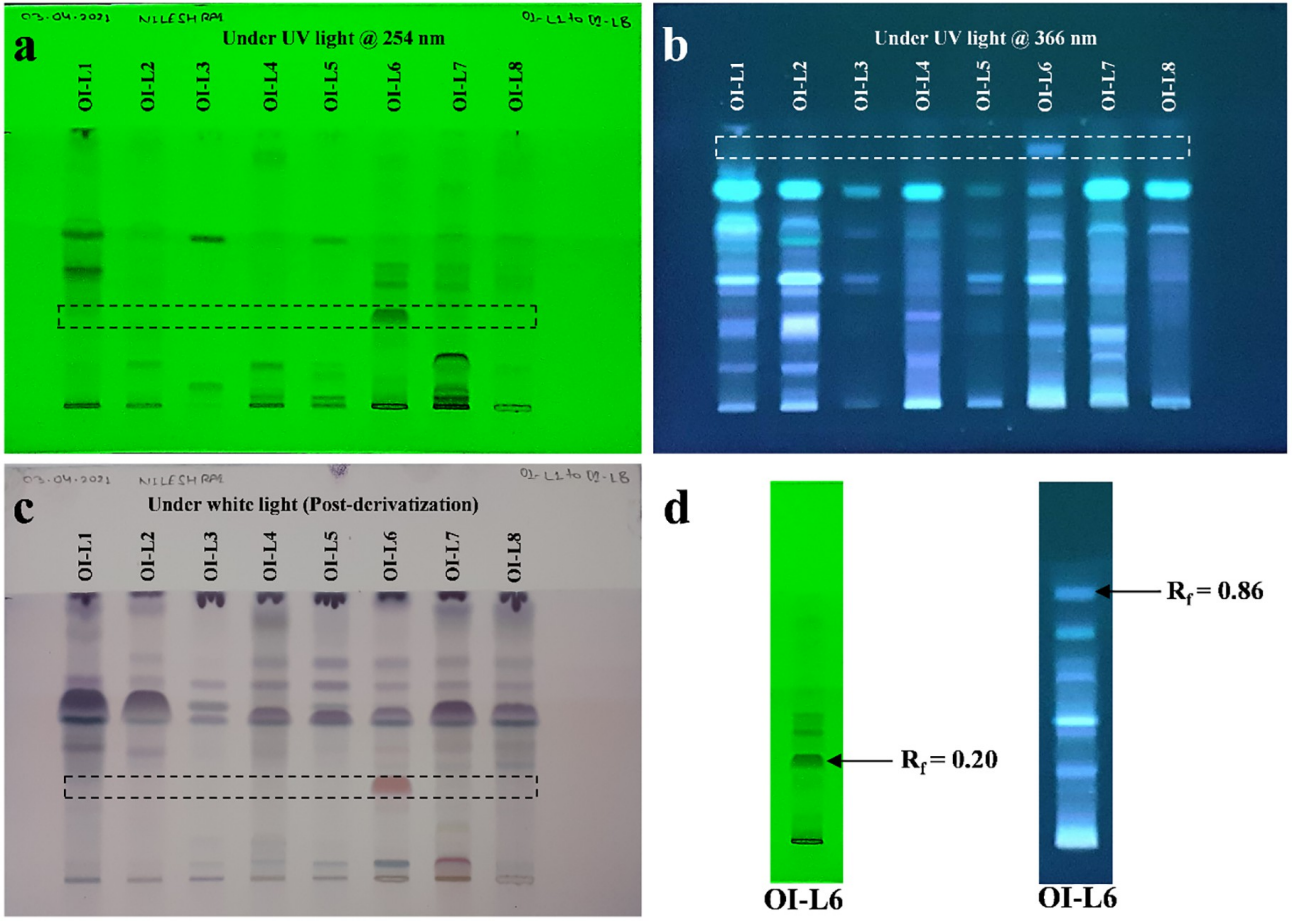
HPTLC fingerprint profiling of EA extract of fungal endophytes **(a)** TLC plate scanned at 254 nm, **(b)** TLC plate scanned at 366 nm, **(c)** TLC plate under white light after derivatization with anisaldehyde sulphuric acid reagent and **(d)** HPTLC fingerprint profiling of EA extract of fungal endophyte *C*. *gloeosporioides* showing unique bioactive component with respective value of retention factor (R_f_).

The HPTLC fingerprinting analysis of EA extract of all identified fungal strains was carried out for the presence of bioactive components. TLC plate scanned at 254 nm (Fig 3a) for fungal strain OI-L1 revealed the presence of 3 major bioactive components at R_f_ values of 0.43, 0.54 and 0.85. HPTLC fingerprint scanned for fungal strain OI-L2 revealed the presence of two major bioactive components at R_f_ values of 0.56 and 0.81 with area percentage of 24.51% and 40.20%, respectively. Similarly, EA extract of fungal strains OI-L3, OI-L4 and OI-L5 showed one major bioactive component for each at R_f_ values of 0.53, 0.81 and 0.55, respectively. OI-L7 and OI-L8 showed the presence of four major bioactive components. Scanning for EA extract of fungal strain OI-L6 revealed the presence of five major bioactive components at R_f_ values of 0.20, 0.38, 0.46, 0.56 and 0.75 with area percentage 26.70%, 13.92%, 16.62%, 13.54% and 8.17%, respectively. TLC plate scanned at 366 nm (Fig 3b) for fungal strains OI-L1, OI-L2 and OI-L5 revealed the presence of three major bioactive components. OI-L7 showed one bioactive component at R_f_ value of 0.72 with area percentage of 75.16%. HPTLC fingerprint analysis of fungal strains OI-L3, OI-L4 and OI-L8 showed two major bioactive components for each. Bioactive components of OI-L3 were at R_f_ values of 0.51 and 0.70, and bioactive components of fungal strain OI-L4 were at R_f_ values of 0.18 and 0.70. Similarly, two bioactive components of OI-L8 were present at R_f_ values of 0.58 and 0.73 with area percentage of 16.09% and 70.10%, respectively. Scanning at 366 nm revealed that EA extract of OI-L6 possess seven major bioactive components at R_f_ values of 0.24, 0.30, 0.34, 0.39, 0.52, 0.71 and 0.86 with area percentage of 5.79%, 5.73%, 5.90%, 14.05%, 30.73%, 22.60% and 5.60%, respectively. Post-chromatography derivatization revealed the presence of terpenoid, steroids, sterols and saponins in EA extract of isolated fungal endophytes (Fig 3c). Out of eight isolated fungal strains, OI-L6 showed the presence of more bioactive components.

TLC chromatogram at 254 nm shows that fungal extract of *C*. *gloeosporioides* (OI-L6) produces an aromatic compound, being unique at R_f_ value 0.20 with 26.70% of relative area percentage. Whereas, TLC chromatogram at 366 nm revealed that out of seven components, the bioactive component present at R_f_ value 0.86 was only produced by endophyte *C*. *gloeosporioides* (Fig 3d). Presence of such unique bioactive components may be responsible for the antioxidant and cytotoxic activity of EA extract of *C*. *gloeosporioides*. HPTLC fingerprint profile showed that fungal strain *C*. *gloeosporioides* produces more and some unique bioactive components than rest of the fungal strains. Post-chromatography derivatization is performed to characterize the presence of various bioactive compounds such as phenols, terpenes, carbohydrates/polyols and steroids by producing violet, blue/red colored band in various shades. Blue-violet, red-violet, purple and red color are produced by terpenoid, steroids and phenols after derivatization with ASR. Terpenes and terpenoids are known to be produced from fungal endophytes with antioxidant, cytotoxic and antimicrobial activity [39]. Blue/red, purple and violet color of bands were prominent in the extract of *C*. *gloeosporioides*, thereby showing the presence of terpenoids, phenols and steroids.

### EA extract of OI-L6 shows potential antioxidant activity against synthetically generated free radicals

#### Antioxidant activity against DPPH free radicals

**Fig 4:**
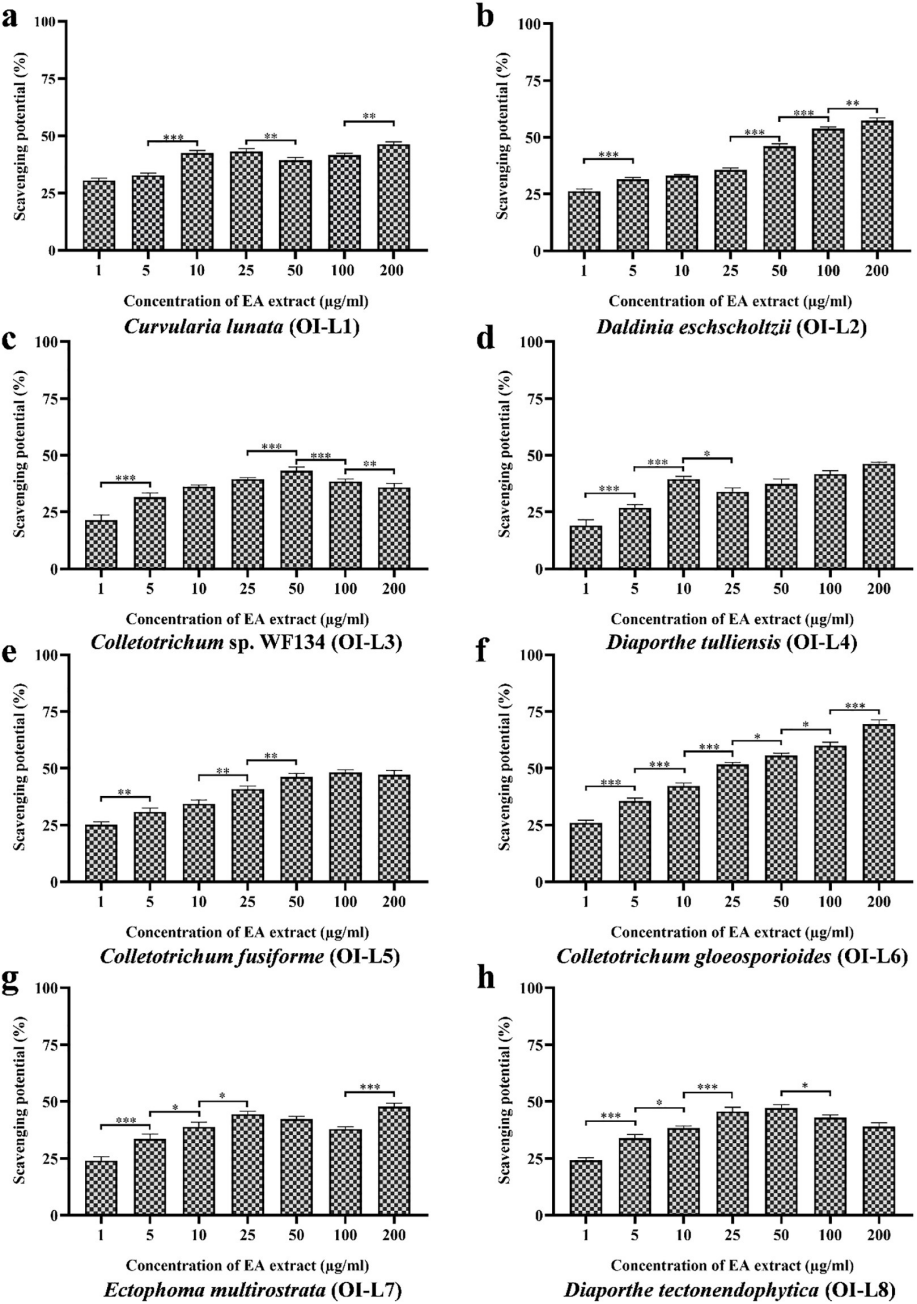
Free radical scavenging potential of EA extract of fungal endophytes **(a)** *C*. *lunata*, **(b)** *D*. *eschscholtzii*, **(c)** *Colletotrichum* sp. WF134, **(d)** *D*. *tulliensis*, **(e)** *C*. *fusiforme*, **(f)** *C*. *gloeosporioides*, **(g)** *E*. *multirostrata* and **(h)** *D*. *tectonendophytica*. All the experiments were performed in triplicate. P-value was calculated by comparing means ± SD of the free radical scavenging potential (%), using one-way ANOVA followed by Tukey to determine statistical significance. Statistical significance are as follows; ***, P≤0.001; **, P ≤0.002; *, P ≤0.033.

The antioxidant activity of EA extract of isolated fungal endophyte was evaluated against the synthetic free radical generated by DPPH (Fig 4). The concentration of EA extract of fungal strains that gives half-maximal response (EC_50_ value) against free radical of DPPH was determined (Table 2). Among the EA extract of eight fungal endophytes, OI-L6 (*C*. *gloeosporioides*) showed the best antioxidant activity with an EC_50_ value of 22.24±1.302 μg/mL. EA extract of OI-L2 (*D*. *eschscholtzii*) also showed moderate antioxidant activity with an EC_50_ value of 74.99±1.108 μg/mL. EA extract of other six fungal endophytes OI-L1 (*C*. *lunata*), OI-L3 (*Colletotrichum* sp. WF134), OI-L4 (*D*. *tulliensis*), OI-L5 (*C*. *fusiforme*), OI-L7 (*E*. *multirostrata*) and OI-L8 (*D*. *tectonendophytica*) were not be able to scavenge free radical of DPPH within the tested range of concentrations thus, possessed EC_50_ value more than 200 μg/mL.

**Table 2:** Antioxidant activity of EA extract of identified fungal endophytes associated with the leaf of *O*. *indicum*.

Isolated strains	Species	EC_50_ value (μg/mL)			
		DPPH free radical scavenging assay	Superoxide anion scavenging activity	Nitric oxide scavenging assay	Hydroxyl radical scavenging assay
OI-L1	*C*. *lunata*	>200	>200	>200	>200
OI-L2	*D*. *eschscholtzii*	74.99±1.108	122.32±0.888	113.77±1.365	110.71±0.918
OI-L3	*Colletotrichum* sp. WF134	>200	>200	>200	>200
OI-L4	*D*. *tulliensis*	>200	133.11±1.356	>200	>200
OI-L5	*C*. *fusiforme*	>200	>200	>200	>200
OI-L6	*C*. *gloeosporioides*	22.24±1.302	67.46±0.576	80.10±0.706	61.55±1.360
OI-L7	*E*. *multirostrata*	>200	>200	>200	>200
OI-L8	*D*. *tectonendophytica*	>200	>200	>200	>200
Ascorbic Acid (Positive control)		11.06±0.372	29.51±3.642	24.46±0.838	18.30±0.907

Oxidative stress imposed by free radicals, reactive oxygen and nitrogen species plays a decisive role in various diseases including cancer. However, the phenolic and flavonoid compounds are reported to show protective activity to the cells by scavenging free radicals [40, 41]. Our results revealed that the EA extract of the fungal endophyte OI-L6 showed significant DPPH free radical scavenging activity in dose-dependent manner when compared with the positive control ascorbic acid (EC_50_ value of 11.06±0.372 μg/mL). Previous report revealed that the crude fungal extract of *C*. *gloeosporioides* isolated from *Justicia gendarussa* exhibits moderate free radical scavenging activity with an IC_50_ value of 135.38 μg/ml that could be validated through the presence of flavonoid content in them [42]. The fungal endophyte, *Colletotrichum gloeosporioides* MKL1, isolated from *Murraya koengii* have shown moderate radical scavenging potential that can be correlated to the flavonoid content [43]. So, the higher free radical scavenging activity of the OI-L6 (*C*. *gloeosporioides*) strain is may be due to the presence of higher phenolic and flavonoid content, which is validated by the content of total phenolics and total flavonoid compounds. The results indicate the higher antioxidant activity of EA extract of *C*. *gloeosporioides*, further suggesting that bioactive compounds produced by *C*. *gloeosporioides* may be a potential candidate to treat certain diseases being either the cause or the consequence of reactive oxygen species.

#### Antioxidant activity against superoxide anion radicals

**Fig 5:**
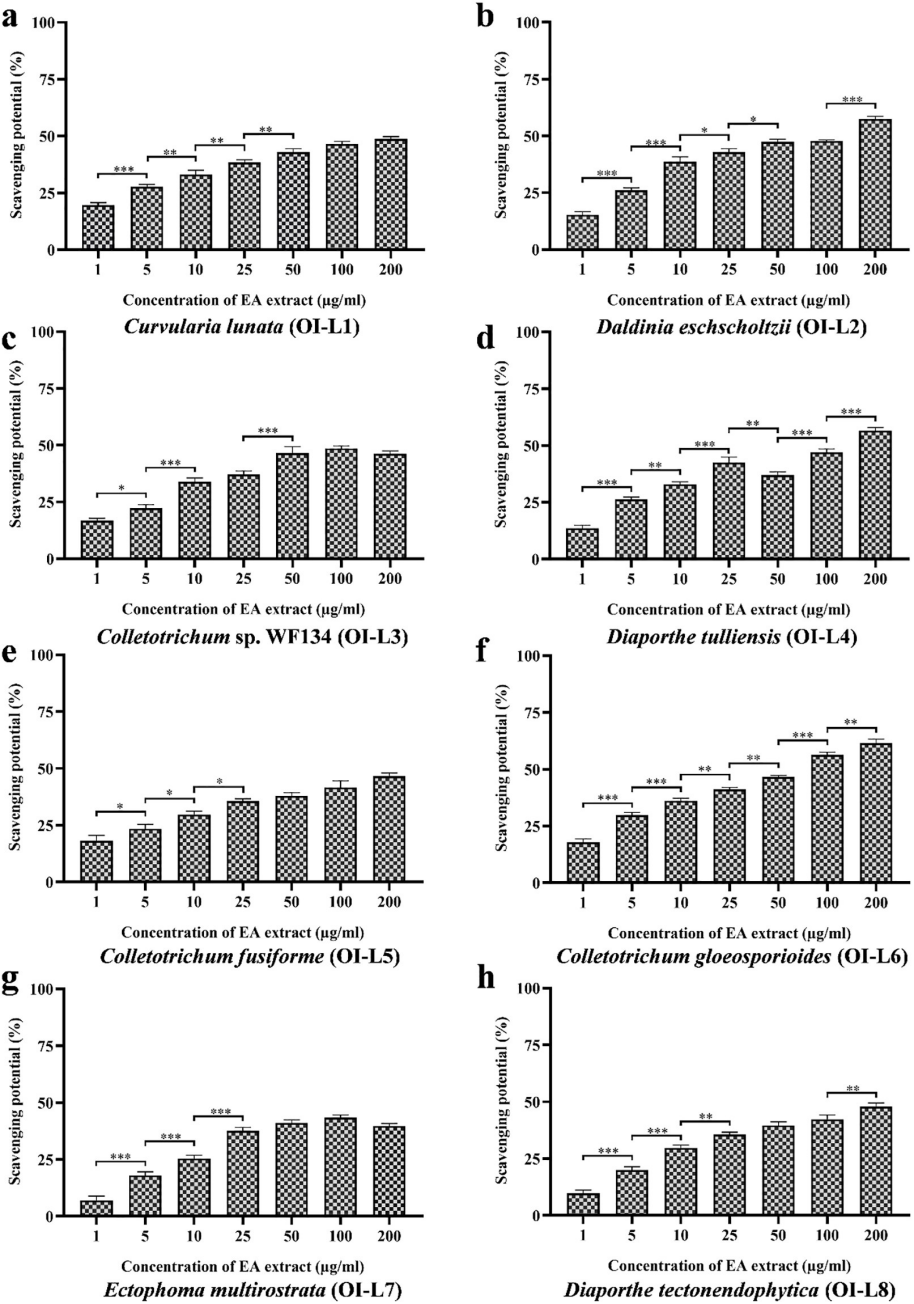
Superoxide anion scavenging potential of EA extract of fungal endophytes **(a)** *C*. *lunata*, **(b)** *D*. *eschscholtzii*, **(c)** *Colletotrichum* sp. WF134, **(d)** *D*. *tulliensis*, **(e)** *C*. *fusiforme*, **(f)** *C*. *gloeosporioides*, **(g)** *E*. *multirostrata* and **(h)** *D*. *tectonendophytica*. All experiments were performed in triplicate. P-value was calculated by comparing means ± SD of the superoxide radical scavenging potential (%), using one-way ANOVA followed by Tukey to determine statistical significance. Statistical significance are as follows; ***, P≤0.001; **, P ≤0.002; *, P ≤0.033.

Superoxide anion assay was performed to evaluate the superoxide anion scavenging potential of EA extract of isolated fungal endophytes (Fig 5). EA extract of endophyte OI-L6 (*C*. *gloeosporioides*) showed the best antioxidant activity as compared to other fungal strains with an EC_50_ value of 67.46±0.576 μg/mL while the EC_50_ value of ascorbic acid was 29.51±3.642 μg/mL (Table 2). EA extract of OI-L2 (*D*. *eschscholtzii*) and OI-L4 (*D*. *tulliensis*) showed moderate superoxide anion scavenging activity with EC_50_ values of 122.32±0.888 μg/mL and 133.11±1.356 μg/mL, respectively. Besides the above strains, EA extract of fungal endophytes OI-L1 (*C*. *lunata*), OI-L3 (*Colletotrichum* sp. WF134), OI-L5 (*C*. *fusiforme*), OI-L7 (*E*. *multirostrata*) and OI-L8 (*D*. *tectonendophytica*) possessed weak super oxide scavenging potential with EC_50_ value more than 200 μg/mL.

In biological system, superoxide anion radicals are known to impose detrimental effect at cellular level [44]. Therefore, it was aimed to evaluate the ability of EA extract of fungal endophytes to scavenge the superoxide anion radicals. Our result reveals that EA extract of OI-L6 exhibited significant scavenging activity against superoxide anion radicals. EA extract of OI-L2 (*D*. *eschscholtzii*) and OI-L4 (*D*. *tulliensis*), also showed moderate scavenging activity but less than that of OI-L6. Several studies have reported a positive link between the presence of flavonoids content and superoxide scavenging property [45, 46]. In our study, total flavonoids content of OI-L6 is maximum as compared to all strains and can be one of the reasons for its high superoxide anion scavenging property.

#### Antioxidant activity against nitric oxide radicals

**Fig 6:**
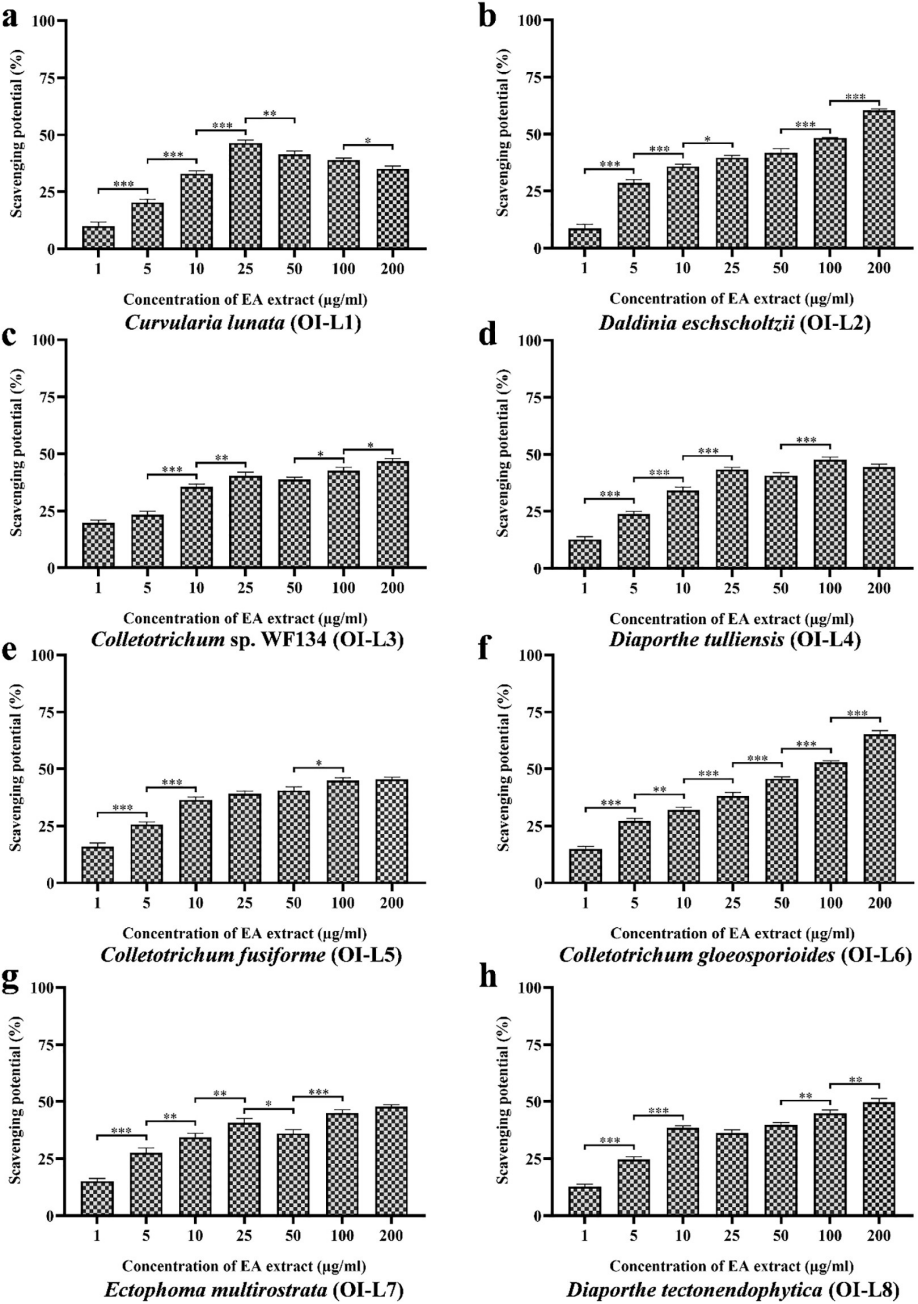
Nitric oxide scavenging potential of EA extract of fungal endophytes **(a)** *C*. *lunata*, **(b)** *D*. *eschscholtzii*, **(c)** *Colletotrichum* sp. WF134, **(d)** *D*. *tulliensis*, **(e)** *C*. *fusiforme*, **(f)** *C*. *gloeosporioides*, **(g)** *E*. *multirostrata* and **(h)** *D*. *tectonendophytica*. All the experiments were performed in triplicate. P-value was calculated by comparing means ± SD of the nitric oxide radical scavenging potential (%), using one-way ANOVA followed by Tukey to determine statistical significance. Statistical significance are as follows; ***, P≤0.001; **, P ≤0.002; *, P ≤0.033.

Nitric oxide scavenging activity was performed to evaluate the scavenging potential of EA extract of fungal endophytes against synthetically generated nitric oxide radicals (Fig 6). EA extract of OI-L6 (*C*. *gloeosporioides*) showed maximal effective response against nitric oxide radicals with an EC_50_ value of 80.10±0.706 μg/mL as compared to other fungal strain and, the EC_50_ value of ascorbic acid (positive control) was estimated to be 24.46±0.838 μg/mL (Table 2). EA extract of OI-L2 (*D*. *eschscholtzii*) exhibited moderate nitric oxide scavenging activity with an EC_50_ value of 113.77±1.365 μg/mL. EA extract of fungal endophytes OI-L1 (*C*. *lunata*), OI-L3 (*Colletotrichum* sp. WF134), OI-L4 (*D*. *tulliensis*), OI-L5 (*C*. *fusiforme*), OI-L7 (*E*. *multirostrata*), and OI-L8 (*D*. *tectonendophytica*) were not be able to scavenge nitric oxide radical within the tested range of concentrations thus, possessed EC_50_ value more than 200 μg/mL.

Nitric oxide radicals are responsible for the various malignancies and inflammatory diseases such as arthritis, multiple sclerosis and juvenile diabetes [47]. Considering the aforementioned facts, the nitric oxide scavenging activity was estimated for the isolated fungal strains. Our result shows that EA extract of OI-L6 showed significant nitric oxide scavenging activity as compared to ascorbic acid, while OI-L2 showed moderate nitric oxide scavenging activity. Thus, EA extract of fungal endophyte OI-L6 can play a significant role in preventing DNA deamination and DNA damage, and may exhibit anticancer activity.

#### Antioxidant activity against hydroxyl radicals

**Fig 7:**
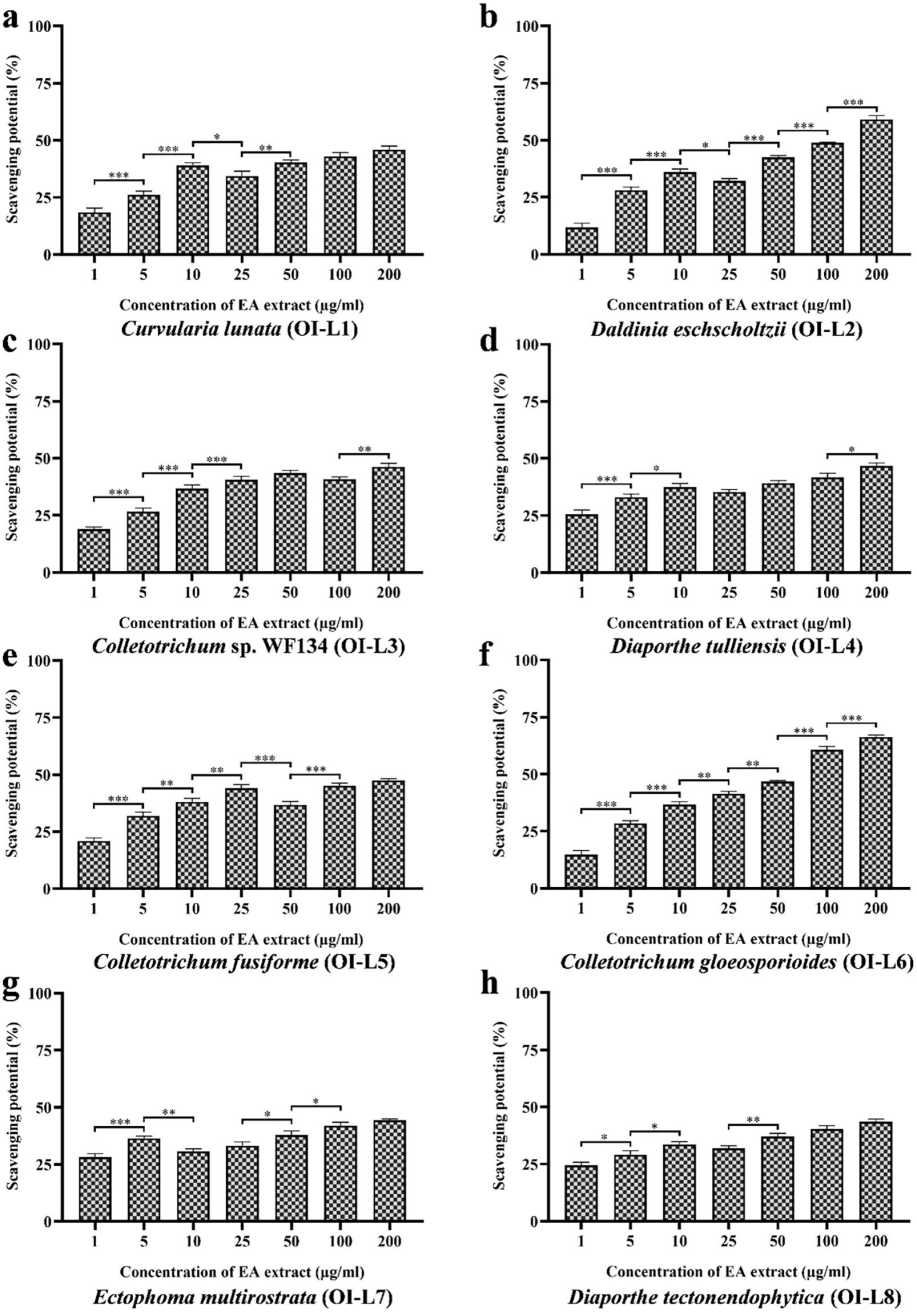
Hydroxyl radical scavenging potential of EA extract of fungal endophytes **(a)** *C*. *lunata*, **(b)** *D*. *eschscholtzii*, **(c)** *Colletotrichum* sp. WF134, **(d)** *D*. *tulliensis*, **(e)** *C*. *fusiforme*, **(f)** *C*. *gloeosporioides*, **(g)** *E*. *multirostrata* and **(h)** *D*. *tectonendophytica*. All experiments were performed in triplicate. P-value was calculated by comparing means ± SD of the hydroxyl anion radical scavenging potential (%), using one-way ANOVA followed by Tukey to determine statistical significance. Statistical significance are as follows; ***, P≤0.001; **, P ≤0.002; *, P ≤0.033.

Hydroxyl radical scavenging activity was performed to evaluate the potential of EA extract of isolated fungal endophytes against hydroxyl radicals (Fig 7). Out of eight fungal strains OI-L6 (*C*. *gloeosporioides*) and OI-L2 (*D*. *eschscholtzii*) showed moderate antioxidant activity against hydroxyl radical with EC_50_ values of 61.55 ±1.360 μg/mL and 110.71±0.918 μg/mL, respectively (Table 2). EA extract of fungal endophytes OI-L1 (*C*. *lunata*), OI-L3 (*Colletotrichum* sp. WF134), OI-L4 (*D*. *tulliensis*), OI-L5 (*C*. *fusiforme*), OI-L7 (*E*. *multirostrata*) and OI-L8 (*D*. *tectonendophytica*) possessed EC_50_ value more than 200 μg/mL. EC_50_ value of ascorbic acid taken as positive control was 18.30±0.907 μg/mL.

Hydroxyl radicals, one of the predominant reactive oxygen species (ROS) which are generated during aerobic metabolism and causes cell damage [48]. Therefore, we performed the hydroxyl radical scavenging potential of EA extract of fungal strains on degradation of 2-deoxyribose which indicates hydroxyl radical scavenging potential. Among all the identified fungal strains, OI-L6 (*C*. *gloeosporioides*) showed the best hydroxyl scavenging property. OI-L2 (*D*. *eschscholtzii*) showed moderate scavenging activity against hydroxyl anion radicals. Collectively, results show that EA extract of eight fungal endophytes have different abilities to scavenge various kind of synthetically generated free radicals which may be attributed to phenolics, flavonoid or other bioactive compounds present in fungal extract. Previous study showed that antioxidant activity of endophyte *Chaetomium* sp. can be attributed to its high phenolic content i.e., 60.13±0.41 mg/GAE [32]. Similar to previous studies, the present study also showed that the fungal isolate OI-L6 (*C*. *gloeosporioides*) produces higher level of phenolic and flavonoid compounds as compared to other fungal isolated fungal isolates, and thereby exhibits significant antioxidant activity by quenching the free radicals, superoxide anion radicals, nitric oxide radicals and hydroxyl radicals than EA extract of other fungal endophytes.

### OI-L6 extract shows significant anti-proliferative activity against cancer cells

**Fig 8:**
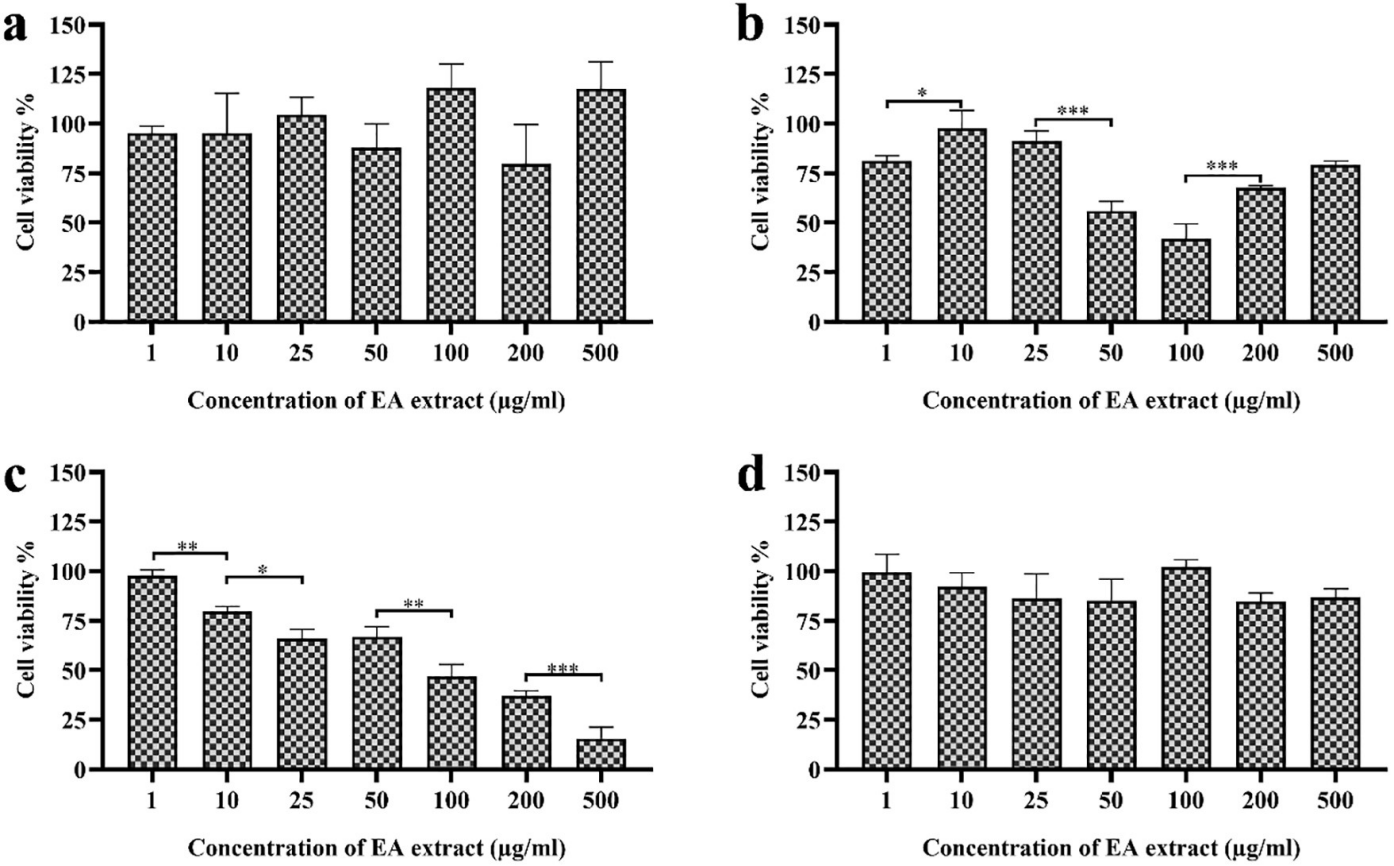
Cytotoxic activity of EA extract of fungal isolates *C*. *gloeosporioides* (OI-L6) against **(a)** HEK 293T cell line, **(b)** HCT 116 cell line, **(c)** HeLa cell line and **(d)** HepG2 cell line. All experiments were performed in triplicate. P-value was calculated by comparing means ± SD of percentage of cell viability of non-cancer and cancer cell, using one-way ANOVA followed by Tukey to determine statistical significance. Statistical significance are as follows; ***, P≤0.001; **, P ≤0.002; *, P ≤0.033.

MTT assay was performed to determine the cytotoxic ability of EA extract of identified fungal endophytes against one non-cancerous cell line (HEK 293T) and three cancerous cell lines (HCT116, HeLa and HepG2 cells) (**S4-S7 Figs in** S1 File) and respective IC_50_ values were shown in (Table 3). Result of MTT assay showed that fungal extract of OI-L7 (*E*. *multirostrata*) and OI-L8 (*D*. *tectonendophytica*) were toxic to non-cancer cells (HEK 293T) at concentrations lower than 2000 μg/mL and were hence not suitable for further study. EA extract of fungal strains OI-L1 (*C*. *lunata*) and OI-L2 (*D*. *eschscholtzii*) were only toxic against human colorectal carcinoma cell line (HCT116) with IC_50_ values of 346.90 μg/mL and 1294 μg/mL, respectively. EA extract of fungal strains OI-L4 (*D*. *tulliensis*) and OI-L5 (*C*. *fusiforme*) were exclusively cytotoxic against HeLa cells with IC_50_ values of 14.90 μg/mL and 390.04 μg/mL, respectively. EA extract of fungal strain OI-L3 (*Colletotrichum* sp. WF134) showed significant toxicity against HeLa and HCT116 cell lines with IC_50_ values of 17.43 μg/mL and 17.56 μg/mL, respectively while, non-toxic against human liver carcinoma cells (HepG2). EA extract of fungal strain OI-L6 was found to exhibit potential cytotoxicity against all the tested cancer cell lines (Fig 8). *C*. *gloeosporioides* showed cytotoxicity against HCT116, HeLa and HepG2 cells with IC_50_ values of 76.59 μg/mL, 176.20 μg/mL and 1750.70 μg/mL, respectively.

**Table 3:** Cytotoxic activity of EA extracts of eight fungal endophytes associated with the leaf of *O*. *indicum* against non-cancer and cancer cell lines.

Isolated strains	Species	IC_50_ value (μg/mL)			
		Non-cancer cell line (HEK 293T)	Colorectal cancer cell (HCT116)	Metastatic breast cancer cell (HeLa)	Liver cancer cell (HepG2)
OI-L1	*C*. *lunata*	>2000	346.90	>2000	>2000
OI-L2	*D*. *eschscholtzii*	>2000	1294	>2000	>2000
OI-L3	*Colletotrichum* sp. WF134	>2000	17.56	17.43	>2000
OI-L4	*D*. *tulliensis*	>2000	>2000	14.90	>2000
OI-L5	*C*. *fusiforme*	>2000	>2000	390.04	>2000
OI-L6	*C*. *gloeosporioides*	1971.15	76.59	176.20	1750.70
OI-L7	*E*. *multirostrata*	169.70	>2000	393.22	>2000
OI-L8	*D*. *tectonendophytica*	11.47	>2000	147.32	213.48
Doxorubicin (Known chemical drug)		-	201.93 μM	0.21 μM	1.25 μM

Need of naturally produced bioactive compounds in the management of cancer is due to adverse side effects of chemotherapeutic drugs, high risk of multi-drug resistance (MDR) and high-cost treatment. Fungal endophytes are potential source of natural bioactive compounds with anticancer property. Previously, various studies have been performed on fungal endophytes to explore the potential bioactive compounds for the management of cancer [49, 50]. Therefore, the EA extract of isolated fungal strains was tested under *in vitro* conditions for cytotoxic activity against different human cancer cells and found to exhibit cancer cell specific cytotoxicity. EA extract of *Colletotrichum* sp. WF134 was cytotoxic specifically against metastatic breast cancer cell line (HeLa) and human colorectal carcinoma cell line (HCT116). Non-toxicity of *Colletotrichum* sp. WF134 extract against human liver carcinoma cells (HepG2) was may be due to the high metabolic rate of liver cancer cells. Similarly, *D*. *tulliensis* showed potent cytotoxicity specifically against HeLa cells. Further, research work is required on multiple cell lines of these cancers to validate the efficacy of EA extract of endophytes *Colletotrichum* sp. WF134 and *D*. *tulliensis* against which, they showed potential cytotoxicity. The EA extract of fungal endophyte *C*. *gloeosporioides* showed moderate to good cytotoxic activity against HCT116, HeLa and HepG2 with IC_50_ values of 76.59 μg/mL, 176.20 μg/mL and 1750.70 μg/mL, respectively. Since, liver is the most common secondary site of metastatic cancer with higher metabolic rate [51]. Therefore, the fungal endophyte derived bioactive compound must possess the cytotoxicity against liver cancer cells. In our study, only the EA extract of endophyte *C*. *gloeosporioides* exhibited cytotoxicity against liver cancer cells HepG2. Thus, it is hypothesized that EA extract of endophyte *C*. *gloeosporioides* has certain bioactive compounds that remain unmetabolized and showed cytotoxicity against HepG2 cells. The cytotoxic activity of EA extract of fungal isolates can be attributed to their significant antioxidant activity. Abnormally high concentration of free radicals in the body can impose hazardous effects to the major cellular constituents including DNA, protein and cell membranes. Oxidative stress is the major cause for the pathogenesis of several disorders including cancer. Carcinogenesis is a multistep process that is initiated with the damage to the nucleic acids, proteins and lipids that further expand to form abnormal cells and ultimately develop into malignant cells upon progression [52]. The antioxidants neutralize the harmful effects of free radicals and thereby prevent development of cancer. EA extract of *C*. *gloeosporioides* showed significantly higher antioxidant activity than EA extract of other isolates, thereby validating the potential cytotoxic activity against the tested cancer cell lines. In previous study, a positive correlation has been established between antioxidant and cytotoxic activity of *Crescentia cujete* L. associated fungal endophyte *Beauveria bassiana*. Among four isolated fungal endophytes, *Beauveria bassiana* exhibited highest antioxidant activity at 13 to 46% and maximum cytotoxicity against human liver cancer cell line (HepG2) with an IC_50_ value of 158.50 μg/ml [53]. The correlation between antioxidant activity and cytotoxicity activity is supported by a study that reports fungal endophytes *Aspergillus fumigatus* and *Fusarium* sp. as most promising amongst six endophytes isolated from *Garcinia* spp. The higher antioxidant activity was reported for *Fusarium* sp. and *Aspergillus fumigatus* with radical scavenging potential of 72.42±2.75% and 60.62±1.10%, respectively. The cytotoxic activity of fungal endophytes against HeLa cell lines showed linear correlation with the antioxidant activity with an IC_50_ value of 92.20±0.23 and 88.54±1.23 μg/ml for the extract of *Fusarium* sp. and *Aspergillus fumigatus*, respectively [54]. Similar to previous reports, our finding also shows a positive correlation between antioxidant and cytotoxicity activity of EA extract of *C*. *gloeosporioides*.

### Comparative analysis between EA extract of OI-L6 and *C*. *gloeosporioides* (MCC 9008) from dead twigs reveals the presence of more bioactive components in OI-L6

**Fig 9:**
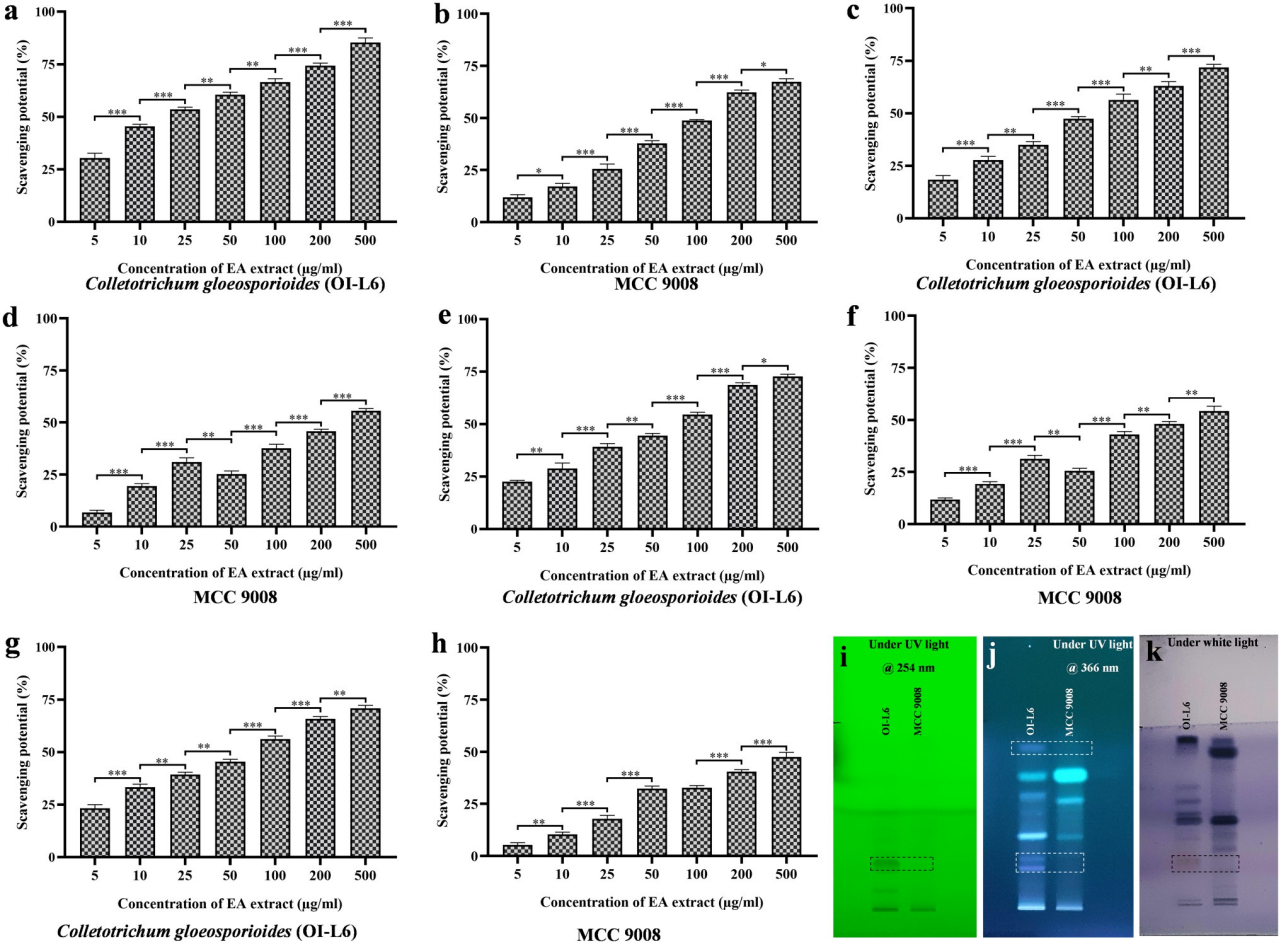
Comparative antioxidant activity and HPTLC fingerprint profiling of EA extract of *C*. *gloeosporioides*. **(a, b)** Free radical scavenging potential of OI-L6 and MCC 9008, **(c, d)** Superoxide anion scavenging potential of OI-L6 and MCC 9008, **(e, f)** Nitric oxide scavenging potential of OI-L6 and MCC 9008, **(g, h)** Hydroxyl radical scavenging potential of OI-L6 and MCC 9008, (**i)** TLC plate scanned at 254 nm, **(j)** TLC plate scanned at 366 nm, **(k)** TLC plate under white light after derivatization with anisaldehyde sulphuric acid reagent. MCC 9008: *C*. *gloeosporioides*, associated with dead twigs. Experiments of antioxidant assays were performed in triplicate. P-value was calculated by comparing the means ± SD of scavenging potential (%) of EA extract of OI-L6 and MCC 9008, using one-way ANOVA followed by Tukey to determine statistical significance. Statistical significance are as follows; ***, P≤0.001; **, P ≤0.002; *, P ≤0.033.

A study to compare the efficacy, antioxidant property and HPTLC fingerprinting pattern of the derived bioactive compounds from OI-L6 (*C*. *gloeosporioides*, associated with *O*. *indicum* leaf) was performed. For comparison we have used fungal strain MCC 9008 (*C*. *gloeosporioides*, accession number MT585639) associated with dead twigs of other plant. The consensus DNA sequence of MCC 9008 was identified by comparing the query sequence in NCBI-BLAST and phylogenetic tree analysis confirmed that fungal strain MCC 9008 clustered into the same clade of *C*. *gloeosporioides*, associated with *O*. *indicum* leaf (**S8 Fig in** S1 File). The antioxidant activity of EA extract of both endophytes was evaluated against DPPH free radical, superoxide anion radical, nitric oxide radical and hydroxyl radical (Table 4). The result of DPPH free radical scavenging assay shows EA extract of OI-L6 demonstrated maximum free radical scavenging activity with an EC_50_ value of 18.47±1.762 μg/mL. While, EA extract of MCC 9008 showed moderate scavenging activity with an EC_50_ value of 108.73±2.259 μg/mL. Therefore, *C*. *gloeosporioides* associated with *O*. *indicum* has approximately six times more potency to scavenge DPPH free radicals (Fig 9a and 9b). EA extract of OI-L6 exhibited maximum superoxide anion scavenging activity with an EC_50_ value of 64.18±0.854 μg/mL. While, EA extract of MCC 9008 showed weak superoxide anion radical scavenging activity with an EC_50_ value of 328.73±1.678 μg/mL (Fig 9c and 9d). EA extract of OI-L6 and MCC 9008 showed nitric oxide radical scavenging activity with EC_50_ values of 77.21±3.223 μg/mL and 292.07±2.839 μg/mL, respectively (Fig 9e and 9f). EA extract of OI-L6 showed maximum hydroxyl radical scavenging activity (EC_50_ value 71.12±2.407 μg/mL) than that of EA extract of MCC 9008. Whereas, EA extract of MCC 9008 was not be able to scavenge the hydroxyl radicals within the tested range of concentrations thus, may possessed EC_50_ value of more than 500 μg/mL (Fig 9g and 9h). Therefore, in all four antioxidant assays, EA extract of *C*. *gloeosporioides* (OI-L6) associated with *O*. *indicum* showed significant antioxidant activity than that of *C*. *gloeosporioides* (MCC 9008) associated with dead twig. Our results demonstrate that bioactive compounds derived from OI-L6 show more antioxidant activity as compared to MCC 9008, suggesting fungal endophyte produces bioactive compounds in a host-dependent manner. In order to study the bioactive components produced by OI-L6 and MCC 9008, we further performed HPTLC fingerprinting analysis of EA extract of OI-L6 and MCC 9008. Image of the TLC plates were captured at short UV range 254 nm, long UV range 366 nm and under white light after derivatization (Fig 9i–9k). TLC plate scanned at 254 nm for EA extract of OI-L6 and MCC 9008 revealed the presence of five major bioactive components at different R_f_ values. HPTLC fingerprinting analysis revealed that EA extract of OI-L6 possessed one unique bioactive component at R_f_ value 0.21 with area percentage of 32.68%. HPTLC fingerprint scanned at 366 nm revealed that EA extract of OI-L6 and MCC 9008 possessed seven and two major bioactive components, respectively. Whereas, OI-L6 produced three major bioactive components at R_f_ values of 0.21, 0.27 and 0.80 with area percentage of 10.31%, 6.44% and 9.93%, respectively. Post-chromatography derivatization revealed that intense violet color band indicate the presence of phenolic compounds, which was four in OI-L6 and only two in MCC 9008. A unique blue/red color band was characteristic for amines, aldehydes, ketones, carbohydrates and esters components which was present in OI-L6 at R_f_ value of 0.21. Thus, OI-L6 showed the presence of more bioactive components than that of MCC 9008.

**Table 4:** Comparative antioxidant activity of EA extract of fungal strains *C*. *gloeosporioides* (OI-L6) and MCC 9008.

Fungal strains	Fungal Species	Host plant	EC_50_ value (μg/mL)			
			DPPH free radical scavenging assay	Superoxide anion scavenging activity	Nitric oxide scavenging assay	Hydroxyl radical scavenging assay
OI-L6	*C*. *gloeosporioides*	Leaf of *O*. *indicum*	18.47±1.762	64.18±0.854	77.21±3.223	71.12±2.407
MCC 9008	*C*. *gloeosporioides*	Dead twigs of other plant	108.73±2.259	328.73±1.678	292.07±2.839	>500
Ascorbic Acid (Positive control)			12.55±0.072	27.54±1.312	20.09±0.081	18.09±0.277

In the present study, the result of antioxidant activity revealed that *C*. *gloeosporioides* associated with the leaf of *O*. *indicum* showed maximum antioxidant activity as compared to *C*. *gloeosporioides* associated with dead twig, this may be attributed to factors such as atmospheric moisture and temperature, geographical location of host plant and host genotype which affects the host–endophyte relationship and colonisation of fungal species inside the host tissues [55, 56]. Ultimately, such factor affects the qualitative and quantitative production of bioactive compounds. The life strategy and biochemical armory of fungal endophyte could alter due to the genotype of their host based on which they express either parasitic or mutualistic lifestyle. An imbalance in the nutrient exchange between host and endophyte results in the change in genetic and biochemical attributes of fungal endophyte [57]. The fungal strain *C*. *gloeosporioides* has been reported to show both symbiotic and pathogenic activity in a host dependent manner. The bioactive compounds from *C*. *gloeosporioides* has been tested to show potent Phosphatidylinositol-3-kinase (PI3K) α inhibitory activity [58], monoamine oxidase (MAO) inhibitory activity [59], antibacterial activity [60] and antifungal activity [61]. However, harmful effects are also reported for *C*. *gloeosporioides* includes anthracnose, seedling blight diseases, pre- and postharvest fruit rots and damping-off and blossom [62]. So, the fungal strains associated with host exhibit varying activities and that is strongly driven by genetic, physiochemical and ecological parameters. Furthermore, HPTLC fingerprinting was carried out for qualitative and quantitative screening of bioactive components present in the EA extracts of OI-L6 and MCC 9008. Comparative TLC plate image analysis under 254 nm showed that a dark green band at R_f_ value 0.21 indicates the presence of a unique aromatic compound in EA extract of *C*. *gloeosporioides* (OI-L6) (Fig 9i). The presence of three unique bioactive components at R_f_ value 0.21, 0.27 and 0.80 found in EA extract of *C*. *gloeosporioides* (OI-L6) was revealed by TLC chromatogram scanned at 366 nm (Fig 9j). The significant antioxidant activity of *C*. *gloeosporioides* (OI-L6) is may be due to the presence of such unique bioactive components. Derivatization reveals the presence of terpenoid, steroids and phenols which gives a blue-violet, red-violet and red color band under white light. Antioxidant activity of bioactive compounds is due to the presence of terpenoids and terpenes [39]. Based on antioxidant activity and HPTLC fingerprinting, it can be concluded that the *C*. *gloeosporioides* associated with the leaf of *O*. *indicum* plant produces more and unique bioactive components with significant antioxidant activity in a host dependent manner.

## Conclusion

In the current study, we have isolated and characterized a total of eight fungal endophytes associated with the leaf of *O*. *indicum* plant. TPC and TFC analysis revealed that extract of *C*. *gloeosporioides* contains highest amount of phenolic and flavonoid content. Presence of unique and large number of bioactive components in extract of *C*. *gloeosporioides* was revealed by HPTLC fingerprinting analysis. Among eight fungal endophytes, the EA extracts of *C*. *gloeosporioides* showed the best antioxidant activity against synthetically generated free radicals DPPH, O_2_^−•^, ^•^NO and ^•^OH. The potential cytotoxic activity of *C*. *gloeosporioides* extract was found in HCT116 cells followed by HeLa and HepG2 cell lines. Host-dependent antioxidant activity of *C*. *gloeosporioides* revealed that EA extract of OI-L6 possess better antioxidant activity and is more potent than MCC 9008. Overall, the study shows that EA extract of fungal endophyte *C*. *gloeosporioides* (OI-L6) shows comparatively significant antioxidant and cytotoxic activity over EA extract of other isolated fungal endophytes, and also produces bioactive compounds in host-dependent manner. Further research work is intended towards the characterization of pure compounds with antioxidant and cytotoxic activity. It will be interesting to validate the antioxidant and cytotoxic activity of EA extract of OI-L6 using suitable *in vivo* models, purification, and characterization of EA extract of OI-L6 using activity guided fractionation.

## Supporting information

S1 File (DOCX)
