# Correction: Bioprospecting of fungal endophytes from *Oroxylum indicum* (L.) Kurz with antioxidant and cytotoxic activity

**DOI:** 10.1371/journal.pone.0308935

**Published:** 2024-08-12

**Authors:** Nilesh Rai, Priyanka Kumari Keshri, Priyamvada Gupta, Ashish Verma, Swapnil C. Kamble, Santosh Kumar Singh, Vibhav Gautam

There are errors in the Author Contributions. The correct contributions are:

**Conceptualization:** Vibhav Gautam.

**Data curation:** Nilesh Rai.

**Formal analysis:** Nilesh Rai, Priyanka Kumari Keshri, Priyamvada Gupta, Ashish Verma, Swapnil C. Kamble, Vibhav Gautam.

**Funding acquisition:** Vibhav Gautam.

**Investigation:** Vibhav Gautam.

**Methodology:** Nilesh Rai.

**Project administration:** Vibhav Gautam.

**Supervision:** Vibhav Gautam.

**Validation:** Nilesh Rai, Vibhav Gautam.

**Visualization:** Vibhav Gautam.

**Writing–original draft:** Nilesh Rai.

**Writing–review & editing:** Priyanka Kumari Keshri, Priyamvada Gupta, Ashish Verma, Swapnil C. Kamble, Santosh Kumar Singh, Vibhav Gautam.
