# Supplementary material for: Bioprospecting of fungal endophytes from Oroxylum indicum (L.) Kurz with antioxidant and cytotoxic activity
Source: PLoS One. 2022 Mar 17;17(3):e0264673. doi: 10.1371/journal.pone.0264673 (PMC8929595; doi:10.1371/journal.pone.0264673)
Supplement: S1 File — (DOCX) [file pone.0264673.s001.docx]

**Supporting information:**

**Bioprospecting of fungal endophytes from *Oroxylum indicum* (L.) Kurz with antioxidant and cytotoxic activity**

Nilesh Rai^1^, Priyanka Kumari Keshri^1^, Priyamvada Gupta^1^, Ashish Verma^1^, Swapnil C. Kamble^2^, Santosh Kumar Singh^1^, Vibhav Gautam*^1^

*^1^Centre of Experimental Medicine and Surgery, Institute of Medical Sciences, Banaras Hindu University, Varanasi-221005, India*

*^2^Department of Technology, Savitribai Phule Pune University, Ganeshkhind, Pune 411007, India*

^*^**Corresponding author:**

Dr. Vibhav Gautam

**Address:**

Centre of Experimental Medicine and Surgery,

Institute of Medical Sciences

Banaras Hindu University

Varanasi 221005, India

Telephone: +918860182113

Email: [vibhav.gautam4@bhu.ac.in](mailto:vibhav.gautam4@bhu.ac.in), vibhavgautam16@gmail.com

**Table S1.** Morphological characteristics of fungal endophytes identified through DNA sequencing of ITS region.

| **Sample code** | **Primer** | **Organism identified through BLAST** | **Primer sequence** | **Morphological properties** | **Accession No** | **References** |
| --- | --- | --- | --- | --- | --- | --- |
| OI-L1 | ITS1 &  ITS4 | *C. lunata* | ITS1 5’-TCCGTAGGTGAACCTGCGG-3’  ITS4 5’ TCCTCCGCTTATTGATATGC 3’ | Colonies olive brown, reverse black. Hyphae septate, nodulose conidiophore, ellipsoidal conidia round at the ends. | MT524328 | [1] |
| OI-L2 | ITS1 &  ITS4 | *D. eschscholtzii* | ITS1 5’-TCCGTAGGTGAACCTGCGG-3’  ITS4 5’ TCCTCCGCTTATTGATATGC 3’ | Culture turned from white to smoky grey in the middle with olivaceous tone. Back side of plate appeared dark due to melanized hyphae. Septate hyphae with exudates, aseptate conidia. | KT763042 | [2] |
|  |  | *Nodulisporium* sp. strain MJ37 | ITS1 5’-TCCGTAGGTGAACCTGCGG-3’  ITS4 5’ TCCTCCGCTTATTGATATGC 3’ | Colonies from white to greenish green. Mycelia- white to grey. Hyphae septate, sometimes branched, Conidiophores unicellular. | MT626605 |  |
| OI-L3 | ITS4 &  ITS5 | *Colletotrichum* sp*.* WF134 | ITS4 5’ TCCTCCGCTTATTGATATGC 3’  ITS5 5’GAAGTAAAAGTCGTAACAAGG 3’ | Colonies from white to green. Mycelia- white to grey, Grey, cottony copious cinnamon masses of conidia, smooth-walled conidiophore, hyaline and unbranched. | HQ130691 | [3] |
| OI-L4 | ITS4 &  ITS5 | *D. tulliensis* | ITS4 5’ TCCTCCGCTTATTGATATGC 3’  ITS5 5’GAAGTAAAAGTCGTAACAAGG 3’ | Flat colony, white mycelia in the center, olivaceous grey mycelium in the periphery. Non-septate conidia, rounded, straight or curved ends. | MN911384 | [4] |
| OI-L5 | ITS4 &  ITS5 | *C. fusiforme* | ITS4 5’ TCCTCCGCTTATTGATATGC 3’  ITS5 5’GAAGTAAAAGTCGTAACAAGG 3’ | Colonies turns white to green, Septate mycelia, Conidiophores with basal setae, cylindrical conidia. | MN538245 | [5] |
| OI-L6 | ITS4 &  ITS5 | *C. gloeosporioides* | ITS4 5’ TCCTCCGCTTATTGATATGC 3’  ITS5 5’GAAGTAAAAGTCGTAACAAGG 3’ | Colonies from white to green. Mycelia- white to smoky grey, smooth walled, septate, cottony hyaline cylindrical conidia and rounded both ends. | LC585212 | [6] |
| OI-L7 | ITS4 &  ITS5 | *E. multirostrata* | ITS4 5’ TCCTCCGCTTATTGATATGC 3’  ITS5 5’GAAGTAAAAGTCGTAACAAGG 3’ | Colony of PDA plate- olive-green to dark brown. Mycelia aerial and compact, ellipsoidal, aseptate, hyaline and single celled conidia. | MT635199 | [7] |
| OI-L8 | ITS4 &  ITS5 | *D. tectonendophytica* | ITS4 5’ TCCTCCGCTTATTGATATGC 3’  ITS5 5’GAAGTAAAAGTCGTAACAAGG 3’ | Flat colony, white mycelia in the center, olivaceous grey mycelium in the periphery. Aseptate conidia, hyaline, ellipsoidal, tapering towards the apex. | MT199850 | [4] |

**Supplementary Figure legends:**

**Fig S1.** Morphological characteristics of *C. gloeosporioides* (OI-L6) **(a)** Leaf sample of *O. indicum*, **(b)** Colony of *C. gloeosporioides* on PDA plate after 10 days at 27 ℃, **(c)** Mycelia, **(d)** Conidiogenous cells, **(e)** Germinating conidia and forming appressoria, and **(f)** Appressoria. Scale bar=100 µm.

**Fig S2.** Gel image of the amplified PCR products (in a range of 500-700 bp) of conserved ITS region of isolated fungal endophytes.

**Fig S3.** Calibration curve represents linear relationship between concentration and absorbance **(a)** Calibration curve for Gallic acid (Standard), and **(b)** Calibration curve for Quercetin (Standard).

**Fig S4.** Cytotoxic activity against HEK 293T cells of EA extract of isolated fungal endophytes **(a)** *C. lunata*, **(b)** *D. eschscholtzii*, **(c)** *Colletotrichum* sp. WF134, **(d)** *D. tulliensis*, **(e)** *C. fusiforme*, **(f)** *C. gloeosporioides*, **(g)** *E. multirostrata*, and **(h)** *D. tectonendophytica*. All experiments were performed in triplicate. P-value was calculated by comparing means ± SD of percentage of cell viability of non-cancer cells HEK 293T, using one-way ANOVA followed by Tukey to determine statistical significance. Statistical significance are as follows; ***, P≤0.001; **, P ≤0.002; *, P ≤0.033.

**Fig S5.** Cytotoxic activity against HCT166 cells of EA extract of isolated fungal endophytes **(a)** *C. lunata*, **(b)** *D. eschscholtzii*, **(c)** *Colletotrichum* sp. WF134, **(d)** *D. tulliensis*, **(e)** *C. fusiforme*, **(f)** *C. gloeosporioides*, **(g)** *E. multirostrata*, and **(h)** *D. tectonendophytica*. All experiments were performed in triplicate. P-value was calculated by comparing means ± SD of percentage of cell viability of cancer cells HCT166, using one-way ANOVA followed by Tukey to determine statistical significance. Statistical significance are as follows; ***, P≤0.001; **, P ≤0.002; *, P ≤0.033.

**Fig S6.** Cytotoxic activity against HeLa cells of EA extract of isolated fungal endophytes **(a)** *C. lunata*, **(b)** *D. eschscholtzii*, **(c)** *Colletotrichum* sp. WF134, **(d)** *D. tulliensis*, **(e)** *C. fusiforme*, **(f)** *C. gloeosporioides*, **(g)** *E. multirostrata*, and **(h)** *D. tectonendophytica*. All experiments were performed in triplicate. P-value was calculated by comparing means ± SD of percentage of cell viability of cancer cells HeLa, using one-way ANOVA followed by Tukey to determine statistical significance. Statistical significance are as follows; ***, P≤0.001; **, P ≤0.002; *, P ≤0.033.

**Fig S7.** Cytotoxic activity against HepG2 cells of EA extract of isolated fungal endophytes **(a)** *C. lunata*, **(b)** *D. eschscholtzii*, **(c)** *Colletotrichum* sp. WF134, **(d)** *D. tulliensis*, **(e)** *C. fusiforme*, **(f)** *C. gloeosporioides*, **(g)** *E. multirostrata*, and **(h)** *D. tectonendophytica*. All experiments were performed in triplicate. P-value was calculated by comparing means ± SD of percentage of cell viability of cancer cells HepG2, using one-way ANOVA followed by Tukey to determine statistical significance. Statistical significance are as follows; ***, P≤0.001; **, P ≤0.002; *, P ≤0.033.

**Fig S8.** Phylogenetic tree of fungal endophytes isolated from leaf of *O. indicum* and MCC 9008 (associated with dead twigs of other plant) constructed by maximum likelihood bootstrap (MLBS) method. The sequences were aligned through MUSCLE alignment program and the evolutionary history was inferred by using the Maximum Likelihood bootstrap (MLBS) method and General Time Reversible model.


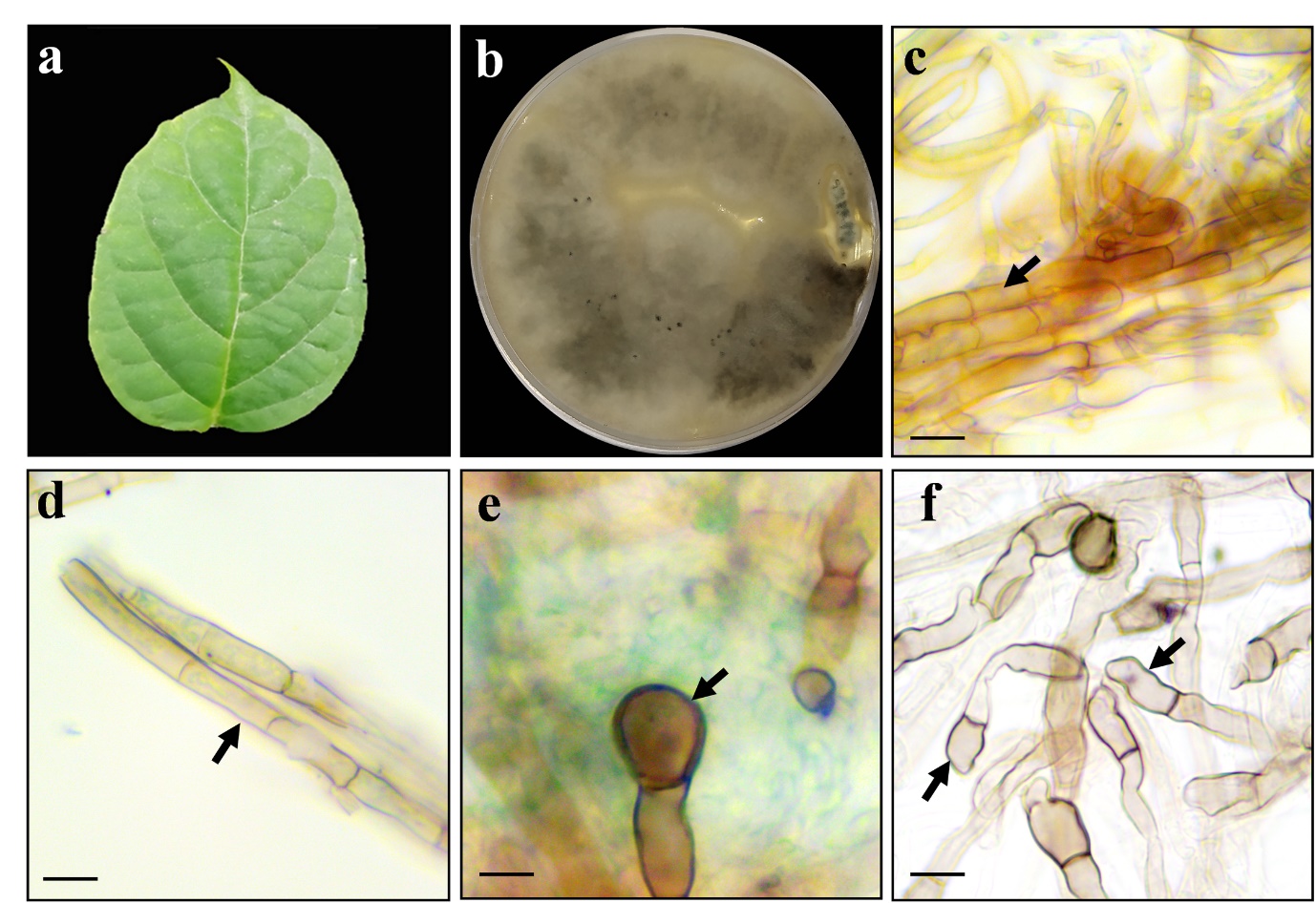


**Fig S1.** Morphological characteristics of *C. gloeosporioides* (OI-L6) **(a)** Leaf sample of *O. indicum*, **(b)** Colony of *C. gloeosporioides* on PDA plate after 10 days at 27 ℃, **(c)** Mycelia, **(d)** Conidiogenous cells, **(e)** Germinating conidia and forming appressoria, and **(f)** Appressoria. Scale bar=100 µm.


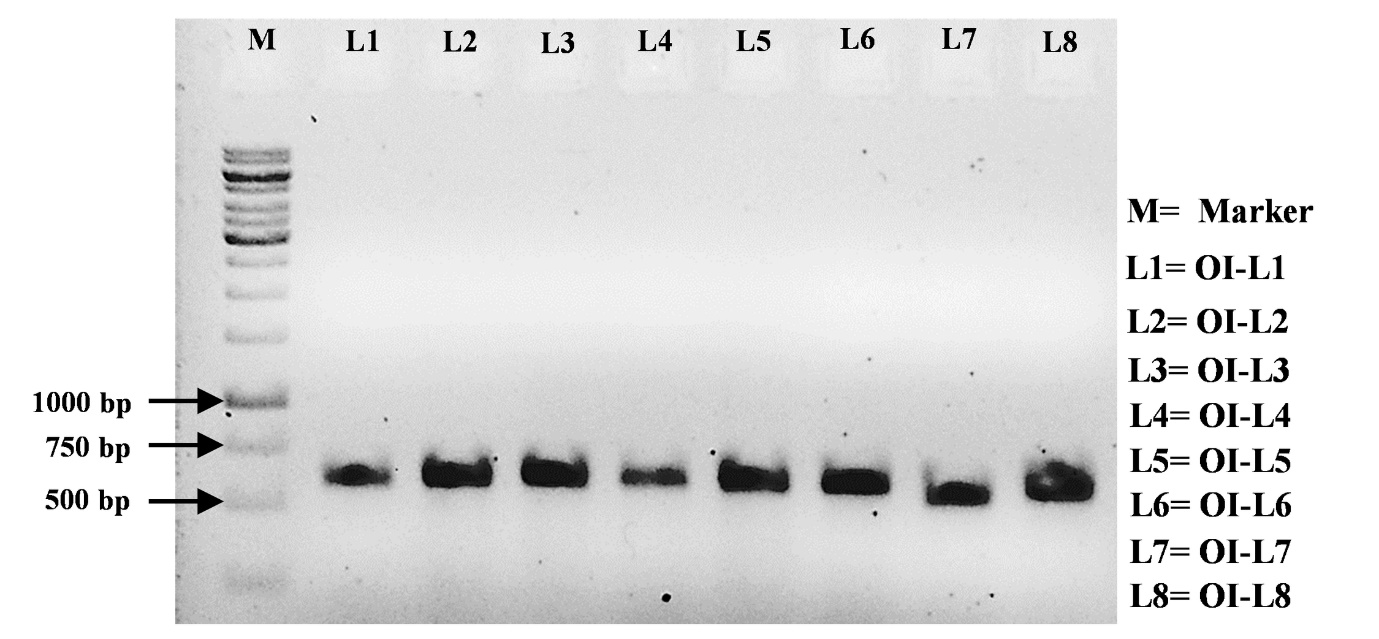


**Fig S2.** Gel image of the amplified PCR products (in a range of 500-700 bp) of conserved ITS region of isolated fungal endophytes.


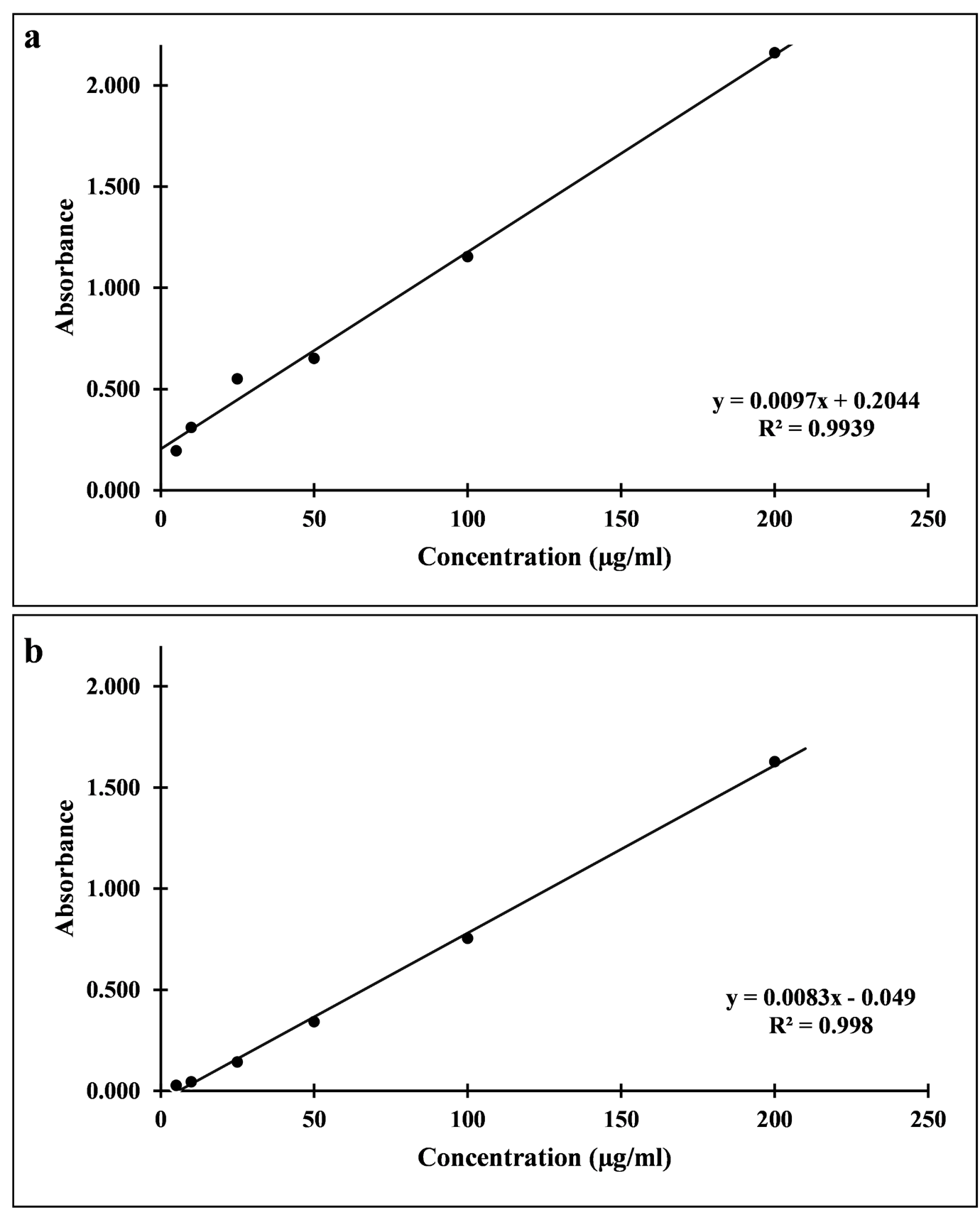


**Fig S3.** Calibration curve represents linear relationship between concentration and absorbance **(a)** Calibration curve for Gallic acid (Standard), and **(b)** Calibration curve for Quercetin (Standard).


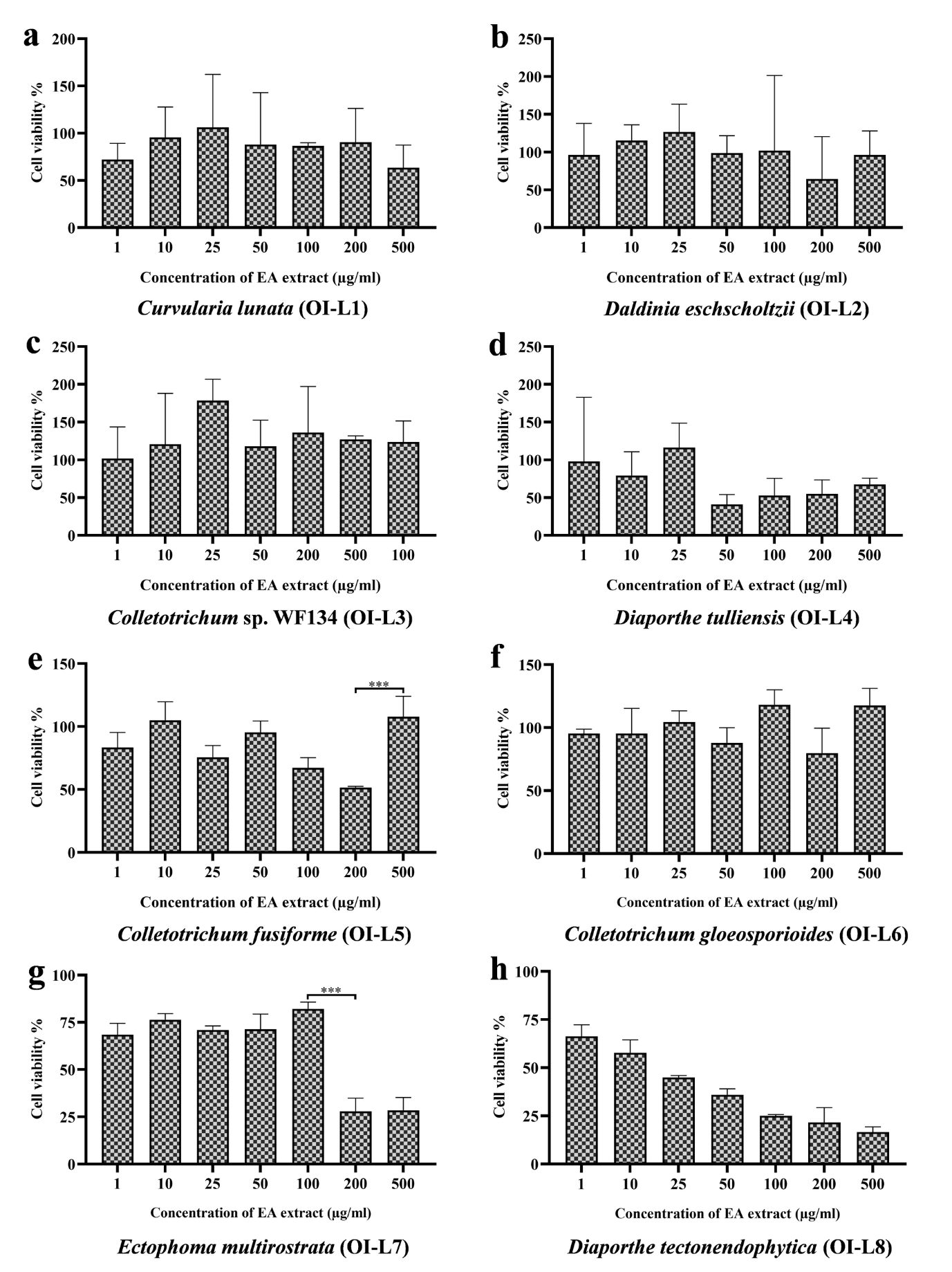


**Fig S4.** Cytotoxic activity against HEK 293T cells of EA extract of isolated fungal endophytes **(a)** *C. lunata*, **(b)** *D. eschscholtzii*, **(c)** *Colletotrichum* sp. WF134, **(d)** *D. tulliensis*, **(e)** *C. fusiforme*, **(f)** *C. gloeosporioides*, **(g)** *E. multirostrata*, and **(h)** *D. tectonendophytica*. All experiments were performed in triplicate. P-value was calculated by comparing means ± SD of percentage of cell viability of non-cancer cells HEK 293T, using one-way ANOVA followed by Tukey to determine statistical significance. Statistical significance are as follows; ***, P≤0.001; **, P ≤0.002; *, P ≤0.033.


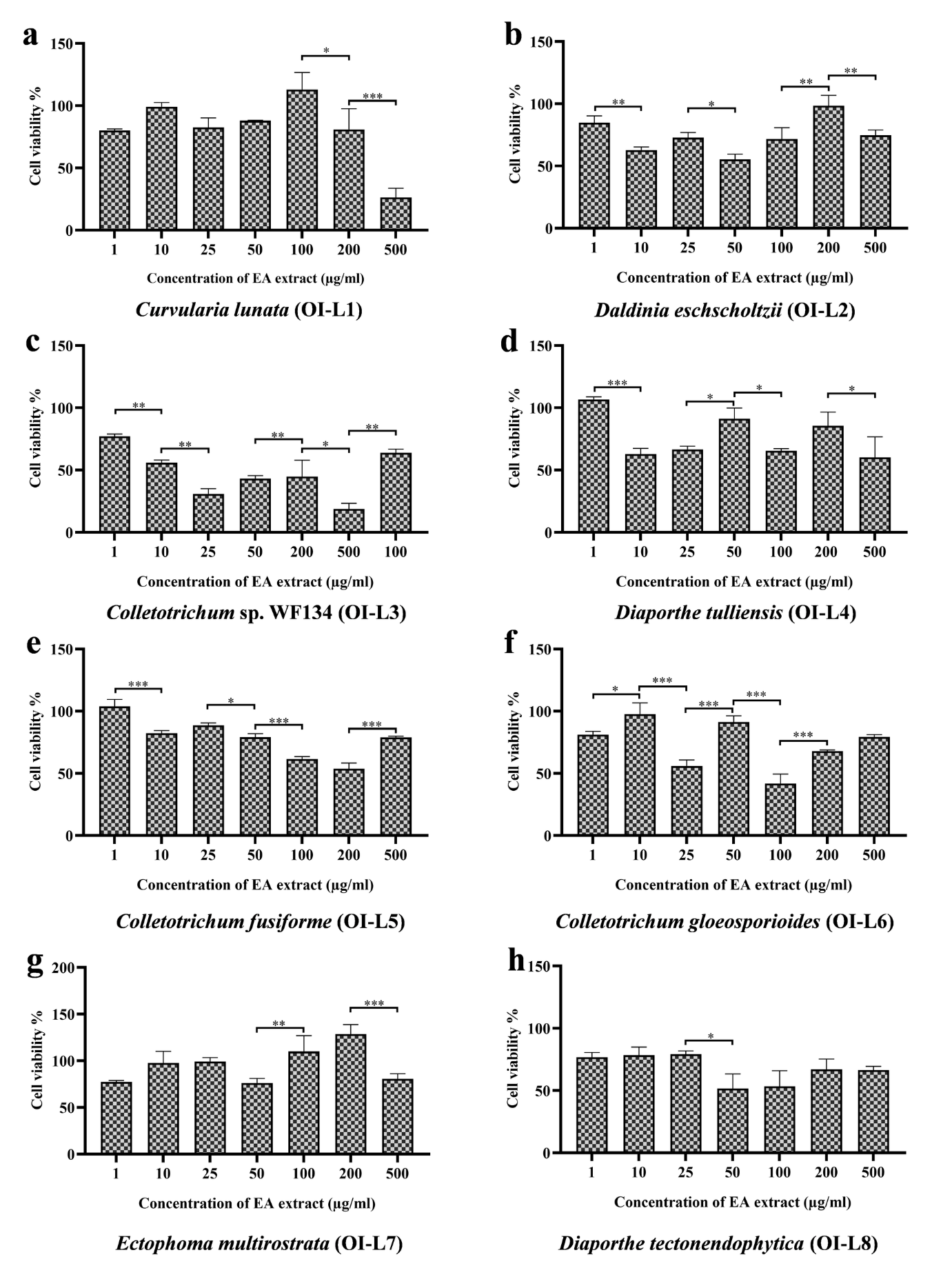


**Fig S5.** Cytotoxic activity against HCT166 cells of EA extract of isolated fungal endophytes **(a)** *C. lunata*, **(b)** *D. eschscholtzii*, **(c)** *Colletotrichum* sp. WF134, **(d)** *D. tulliensis*, **(e)** *C. fusiforme*, **(f)** *C. gloeosporioides*, **(g)** *E. multirostrata*, and **(h)** *D. tectonendophytica*. All experiments were performed in triplicate. P-value was calculated by comparing means ± SD of percentage of cell viability of cancer cells HCT166, using one-way ANOVA followed by Tukey to determine statistical significance. Statistical significance are as follows; ***, P≤0.001; **, P ≤0.002; *, P ≤0.033.


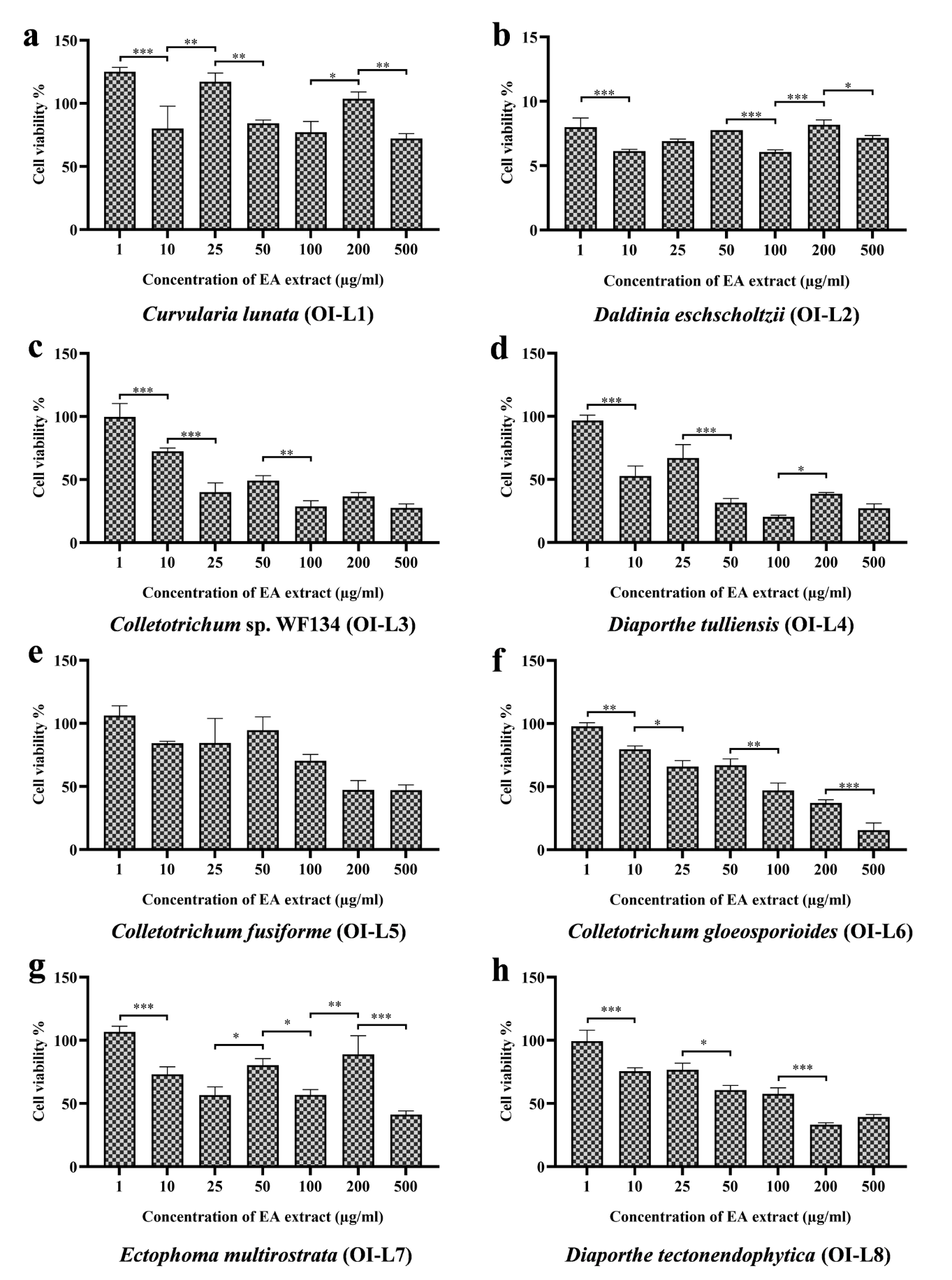


**Fig S6.** Cytotoxic activity against HeLa cells of EA extract of isolated fungal endophytes **(a)** *C. lunata*, **(b)** *D. eschscholtzii*, **(c)** *Colletotrichum* sp. WF134, **(d)** *D. tulliensis*, **(e)** *C. fusiforme*, **(f)** *C. gloeosporioides*, **(g)** *E. multirostrata*, and **(h)** *D. tectonendophytica*. All experiments were performed in triplicate. P-value was calculated by comparing means ± SD of percentage of cell viability of cancer cells HeLa, using one-way ANOVA followed by Tukey to determine statistical significance. Statistical significance are as follows; ***, P≤0.001; **, P ≤0.002; *, P ≤0.033.


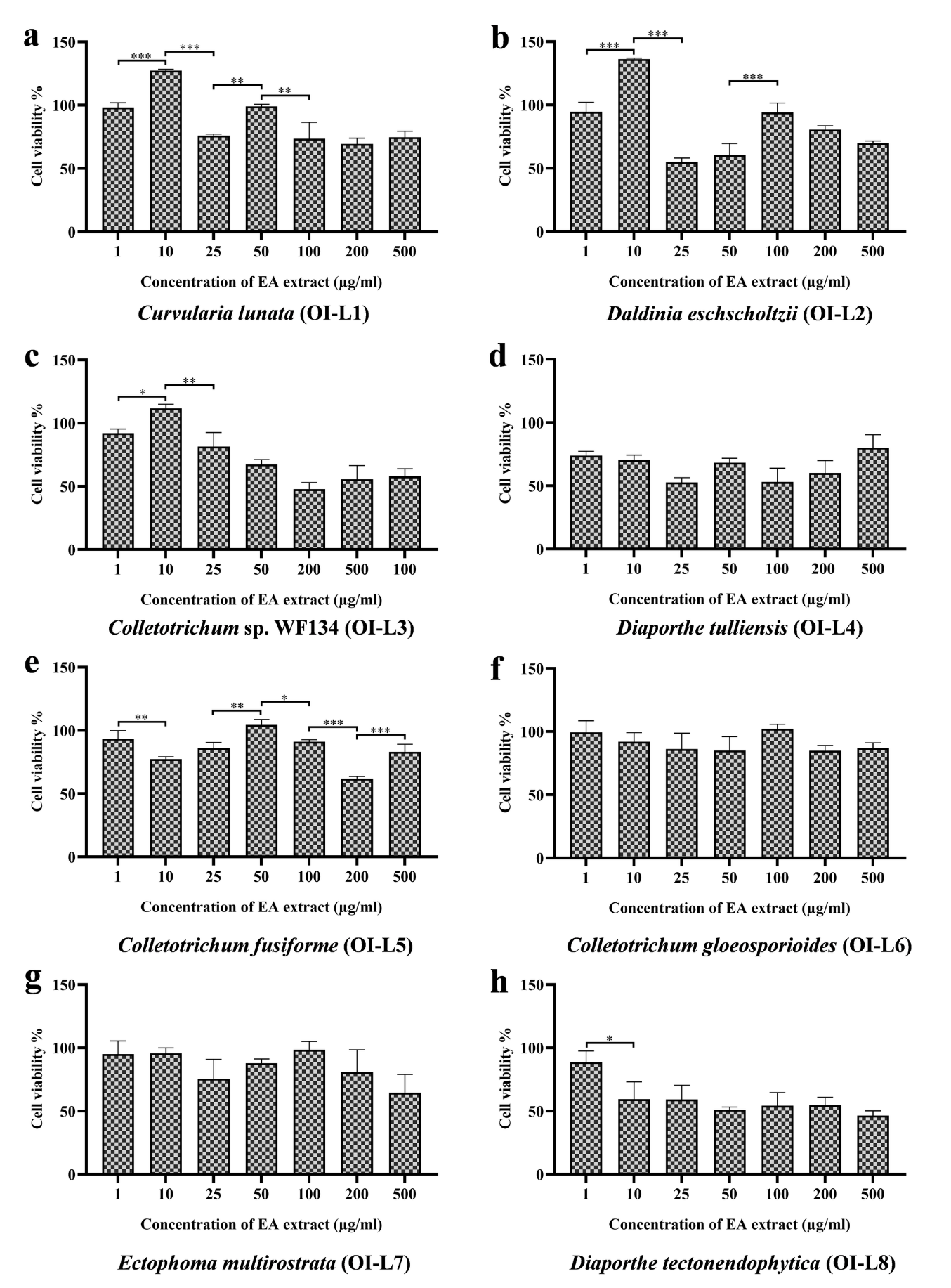


**Fig S7.** Cytotoxic activity against HepG2 cells of EA extract of isolated fungal endophytes **(a)** *C. lunata*, **(b)** *D. eschscholtzii*, **(c)** *Colletotrichum* sp. WF134, **(d)** *D. tulliensis*, **(e)** *C. fusiforme*, **(f)** *C. gloeosporioides*, **(g)** *E. multirostrata*, and **(h)** *D. tectonendophytica*. All experiments were performed in triplicate. P-value was calculated by comparing means ± SD of percentage of cell viability of cancer cells HepG2, using one-way ANOVA followed by Tukey to determine statistical significance. Statistical significance are as follows; ***, P≤0.001; **, P ≤0.002; *, P ≤0.033.


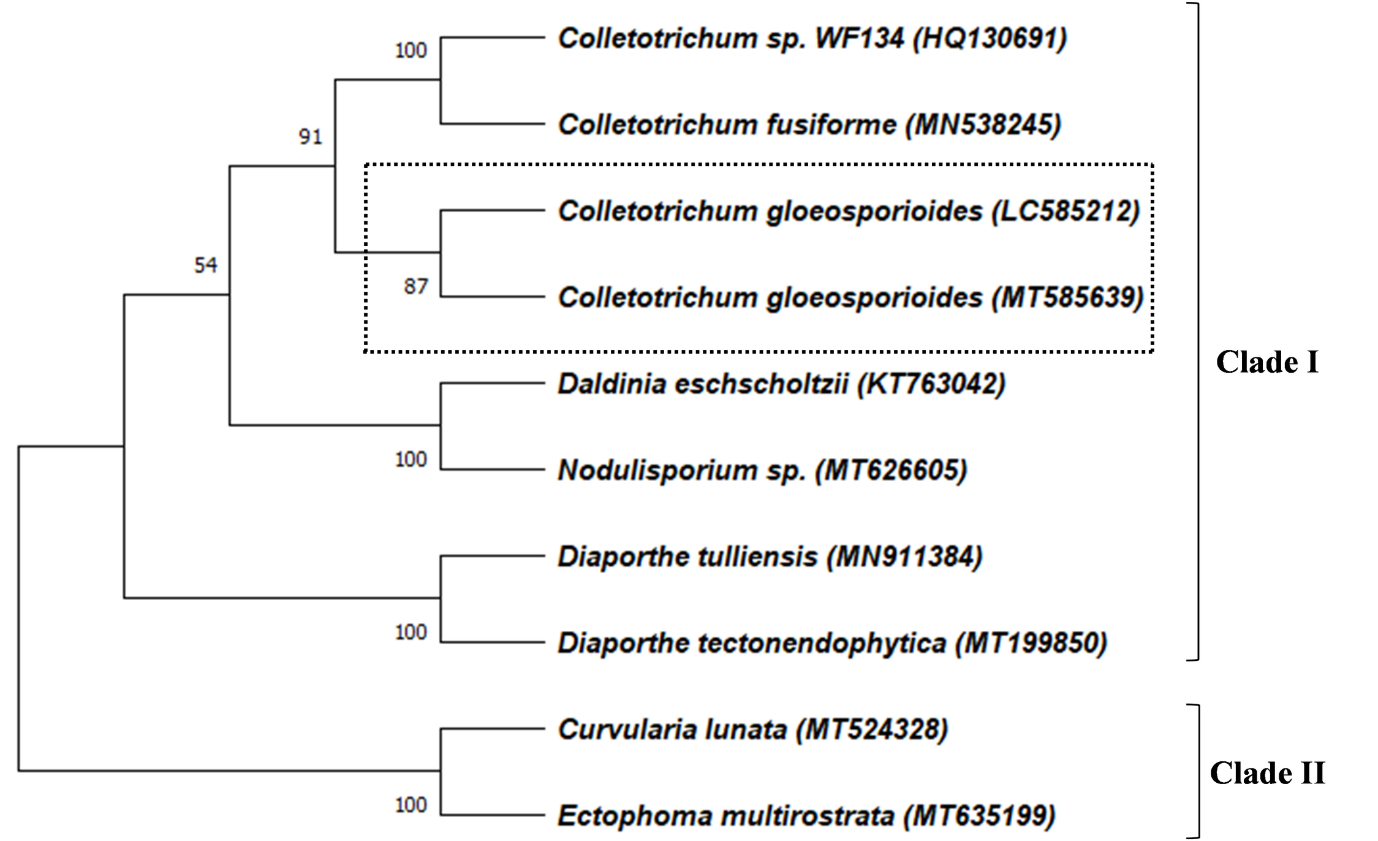


**Fig S8.** Phylogenetic tree of fungal endophytes isolated from leaf of *O. indicum* and MCC 9008 (associated with dead twigs of other plant) constructed by maximum likelihood bootstrap (MLBS) method. The sequences were aligned through MUSCLE alignment program and the evolutionary history was inferred by using the Maximum Likelihood bootstrap (MLBS) method and General Time Reversible model.

**References**

1. Manamgoda DS, Cai L, McKenzie EH, Crous PW, Madrid H, Chukeatirote E, et al. A phylogenetic and taxonomic re-evaluation of the Bipolaris-Cochliobolus-Curvularia complex. Fungal diversity. 2012;56(1):131-44.

2. Stadler M, Læssøe T, Fournier J, Decock C, Schmieschek B, Tichy H-V, et al. A polyphasic taxonomy of Daldinia (Xylariaceae). Studies in mycology. 2014;77:1-143.

3. Ma X, Nontachaiyapoom S, Jayawardena RS. Endophytic Colletotrichum species from Dendrobium spp. in China and Northern Thailand. MycoKeys. 2018;(43):23.

4. Dissanayake A, Phillips A, Hyde K, Yan J, Li X. The current status of species in Diaporthe. Mycosphere. 2017;8(5):1106-56.

5. Samarakoon M, Peršoh D, Hyde K, Bulgakov T, Manawasinghe I, Jayawardena R, et al. Colletotrichum acidae sp. nov. from northern Thailand and a new record of C. dematium on Iris sp. Mycosphere. 2018;9(3):583-97.

6. Weir B, Johnston P, Damm U. The Colletotrichum gloeosporioides species complex. Studies in mycology. 2012;73:115-80.

7. Valenzuela-Lopez N, Cano-Lira J, Guarro J, Sutton DA, Wiederhold N, Crous P, et al. Coelomycetous Dothideomycetes with emphasis on the families Cucurbitariaceae and Didymellaceae. Studies in mycology. 2018;90:1-69.
